# Rhizosphere 16S-ITS Metabarcoding Profiles in Banana Crops Are Affected by Nematodes, Cultivation, and Local Climatic Variations

**DOI:** 10.3389/fmicb.2022.855110

**Published:** 2022-06-09

**Authors:** Aurelio Ciancio, Laura Cristina Rosso, Javier Lopez-Cepero, Mariantonietta Colagiero

**Affiliations:** ^1^Consiglio Nazionale delle Ricerche, Istituto per la Protezione Sostenibile delle Piante, Bari, Italy; ^2^Departamento Técnico de Coplaca S.C., Organización de Productores de Plátanos, Santa Cruz de Tenerife, Spain

**Keywords:** 16S rRNA, *Helicotylenchus multicinctus*, ITS, *Pratylenchus goodeyi*, microbiota

## Abstract

Agriculture affects soil and root microbial communities. However, detailed knowledge is needed on the effects of cropping on rhizosphere, including biological control agents (BCA) of nematodes. A metabarcoding study was carried out on the microbiota associated with plant parasitic and other nematode functional groups present in banana farms in Tenerife (Canary Islands, Spain). Samples included rhizosphere soil from cv Pequeña Enana or Gruesa and controls collected from adjacent sites, with the same agroecological conditions, without banana roots. To characterize the bacterial communities, the V3 and V4 variable regions of the 16S rRNA ribosomal gene were amplified, whereas the internal transcribed spacer (ITS) region was used for the fungi present in the same samples. Libraries were sequenced with an Illumina MiSeq™ in paired ends with a 300-bp read length. For each sample, plant parasitic nematodes (PPN) and other nematodes were extracted from the soil, counted, and identified. Phytoparasitic nematodes were mostly found in banana rhizosphere. They included *Pratylenchus goodeyi*, present in northern farms, and *Helicotylenchus* spp., including *H. multicinctus*, found in both northern and southern farms. Metabarcoding data showed a direct effect of cropping on microbial communities, and latitude-related factors that separated northern and southern controls from banana rizosphere samples. Several fungal taxa known as nematode BCA were identified, with endophytes, mycorrhizal species, and obligate Rozellomycota endoparasites, almost only present in the banana samples. The dominant bacterial phyla were Proteobacteria, Actinobacteria, Planctomycetes, Bacteroidetes, Chloroflexi, and Acidobacteria. The ITS data showed several operational taxonomic units (OTUs) belonging to Sordariomycetes, including biocontrol agents, such as *Beauveria* spp., *Arthrobotrys* spp., *Pochonia chlamydosporia*, and *Metarhizium anisopliae*. Other taxa included *Trichoderma harzianum, Trichoderma longibrachiatum, Trichoderma virens*, and *Fusarium* spp., together with mycoparasites such as *Acrostalagmus luteoalbus*. However, only one *Dactylella* spp. showed a correlation with predatory nematodes. Differences among the nematode guilds were found, as phytoparasitic, free-living, and predatory nematode groups were correlated with specific subsets of other bacteria and fungi. Crop cultivation method and soil texture showed differences in taxa representations when considering other farm and soil variables. The data showed changes in the rhizosphere and soil microbiota related to trophic specialization and specific adaptations, affecting decomposers, beneficial endophytes, mycorrhizae, or BCA, and plant pathogens.

## Introduction

Banana (*Musa acuminata*) is an economically important crop in the Canary Islands (Spain), where it represents a source of income for many small holders, with around 50% of cultivated areas and yields that supply around 70% of national consumption. Dwarf Cavendish (Pequeña Enana, AAA) and derived selections represent the most widespread genotype (>90% of plants) due to its productivity and commercial performance. The crop is cultivated in small parcels, with alkaline volcanic soils often proceeding from different sites, amended with organic matter in part originating from old leaves left rotting on the soil surface. The main adversities of this cultivation include nematode pests, banana weevil (*Cosmopolites sordidus*), and Fusarium wilt caused by *Fusarium oxysporum* f. sp. *cubense* (Foc). The latter disease affects 2–12% of plants, with some farms reaching up to 30% prevalence (Gómez-Lama Cabanás et al., 2021). Pest and Foc management include a conventional approach, based on nematicides and insecticides, pheromone traps or products based on cloripirifos, spirodiclofen, and other active components. Alternative organic approaches for pest and disease management include the use of natural products with nematocidal or fungicidal properties, combined with pheromone traps (Supplementary Table 1).

PPN found in the Canary Islands banana farms include lesion nematodes *Pratylenchus* spp., spiral nematodes *Helicotylenchus* spp., and occasionally root-knot nematodes, *Meloidogyne* spp. These parasites are widespread in banana cultivated areas of the world (Moens et al., 2006; Coyne et al., 2018). Fusarium wilt is the most severe soil-borne vascular disease encountered in the Canary Islands. Its virulence depends on the race as well as on plant tolerance level (Bubici et al., 2019; Gómez-Lama Cabanás et al., 2021). The races reported in the Canary Islands are the subtropical races 4 (SR4), R2, and R1 (vegetative compatibility group 0120), highly virulent on cv. Gros Michel but not on Grand Naine (Domínguez et al., 2001). The low virulence of those races appears to be related to suppressive soil factors, such as the scarcity of iron and high levels of sodium and clay (Domínguez et al., 2001).

Plant and soil microbiota have an important effect on crop productivity. They include species with a varying degree of specialization, underpinning fundamental services such as nutrient recycling and pest or disease regulation. Is it recognized that many species, including BCA, contribute significantly to crop production by sustaining the rhizosphere and plant health (Bulgarelli et al., 2013; Granzow et al., 2017; Jacoby et al., 2017; Berg et al., 2020). The rhizosphere microbiota may hence represent a natural reservoir of BCA and a possible alternative tool for PPN management (Berg et al., 2017). However, due to the complexity of the rhizosphere environment, natural pest/disease regulation in intensive crops is not as frequent as expected. This often occurs in the case of PPN in intensive cropping systems (Topalović and Heuer, 2019; Topalović et al., 2020). The severity of PPN attacks mostly depends on the changes induced in soil by agriculture, such as the environmental pressure of monocultures and/or the low genetic diversity of crops. These conditions occur, and are particularly evident, in banana crops, in which all plants are usually clones of one or few lineages (mostly Cavendish), selected for commercial or technical reasons including resistance to one or more Foc diseases.

Knowledge of soil and rhizosphere microbial diversity and composition is hence fundamental to implement sustainable crop management, as well as to determine the impact of one or more biological/technical factors on the indigenous BCA. Progress has been achieved in determining the links between Fusarium wilt and banana-associated microorganisms (Effendi et al., 2019; Kaushal et al., 2020; Gómez-Lama Cabanás et al., 2021; Ravi et al., 2021). However, the potential of soil microorganisms to manage plant production in a more sustainable way, relying solely on indigenous species, is not yet fully exploited. Intensive banana crop management still remains highly dependent on frequent applications of synthetic pesticides. In particular, it is unknown if and how co-occurring microbial species, as well as their interactions with the BCA present, act on soil pests such as PPN. Soil-inhabiting organisms form a complex food web system, whose final outcomes may range from the natural regulation of noxious organisms to the insurgence of severe pest attacks (Bardgett et al., 1999; Ram et al., 2008).

Soil microbial communities, including BCA with other cryptic co-occurring species, may reveal undetected but useful interactions with the farm and the local agroenvironment (i.e., soil physical properties and pests), or depending on other external variables (i.e., selection of cropping practices and climate). In this regard, belowground links among species and trophic groups are becoming more and more informative as a huge amount of data is made available through deep sequencing and -omics technologies (Berg et al., 2020; Martínez Arbas et al., 2021). A detailed knowledge of soil microbial profiles may allow the setup of information-based crop management practices, exploiting natural pest/disease regulation, reinforcing or sustaining soil, and rhizosphere health.

Beneficial microorganisms that contribute to plant production include species acting as generalists or, on the contrary, inhabiting specific trophic niches, ranging from endophytes to pathogens or BCA, which affect plant health and thus farm productivity (Bulgarelli et al., 2013; Xue et al., 2015; Granzow et al., 2017; Kaushal et al., 2020; Zhang et al., 2020; Gómez-Lama Cabanás et al., 2021; Ravi et al., 2021). Apart from plant growth promoters, soil harbors endosymbionts or BCA often specifically associated with their hosts. Some isolates are already exploited as active ingredients in a number of commercial products, worldwide. These include, for example, host-specific PPN antagonists such as the bacteria *Pasteuria* spp. or *Bacillus* spp. (Tian et al., 2007; Mohan et al., 2020), and ubiquitous fungi such as *Pochonia chlamydosporia*, a root endophyte and a parasite of nematode eggs (Manzanilla-López et al., 2013). As a root endophyte, *P. chlamydosporia* also elicits the expression of a wide range of plant defense genes (Larriba et al., 2015; Mingot-Ureta et al., 2020; Tolba et al., 2021; Zhuang et al., 2021).

Other fungi of interest for exploitation as BCA are the members of genera *Metarhizium* and *Trichoderma*. The former, phylogenetically close to *Pochonia*, is characterized by the root endophytism and soil insect parasitism. Within the genus *Trichoderma*, several BCA and/or endophytes are present, including its teleomorph genus *Hypocrea* (Sivan and Chet, 1986; Chaverri and Samuels, 2002). However, data are needed on the competence and persistence of these BCA in the soil and rhizosphere, in particular when they are naturally present, as well as on their interactions with associated bacteria.

A further factor impacting the rhizosphere environment, and thus natural pest or disease regulation, originates from anthropic activities including, i.e., practices adopted by producers through organic or conventional farming, and the related aboveground cover, which also affect belowground microbiota profiles (Leff et al., 2018). Few data are available on the effect of organic and low-impact agriculture, soil properties, climate, on the microbiota diversity and, indirectly, on BCA species distribution, prevalence, and co-occurrence.

Considering the important role played by soil microorganisms in crop production and pest regulation, the focus of this study was to evaluate the interactions of the banana crop with the rhizosphere microbiota and the indigenous BCA of nematodes present in the soil, also to evaluate additional variables. In this study, we produced and analyzed metabarcoding data to: (1) determine the effect of cropping on rhizosphere bacteria and fungi in banana farms, in a subtropical environment, (2) investigate the effect of farm properties on rhizosphere microbial profiles, (3) estimate the impact that factors, such as climate or soil properties, and nematodes have on soil microbiota profiles, and (4) evaluate the crop effects on naturally occurring BCA species as well as their potential for PPN regulation.

## Materials and Methods

A total of 38 samples were collected in Tenerife (Canary Islands, Spain) from the rhizosphere of *M. acuminata* var. Pequeña Enana or Gruesa (Cavendish clones), on farms applying conventional (only synthetic chemicals), integrated pest management (IPM, using chemical pesticides and natural products), or organic cropping techniques (EU regulation n. 848/2018) (Supplementary Table 1). A total of 36 samples were collected in February 2018 (mean month temperature = 18°C, humidity = 65%). Rhizosphere soil samples (ca. 300 ml) were collected at an average depth of 20 cm with banana root fragments. Other local control samples, with mostly grass or weed roots, were collected on the same farms from adjacent, banana root-free sites, within 5–10 m from the sampled banana plants, at the same depth. The three replicate banana samples and corresponding controls (each formed by three or more nearby subsamples, mixed to form a sample) were collected in six farms, located in the northern and southern areas of the island (Supplementary Table 1). Two other samples from banana rhizosphere, collected from a northern farm in September 2017 and stored at 4°C, were also included in this study. A subsample (300 g of mixed soil and roots) from each soil bag was stored at −80°C before processing for subsequent analyses. The remaining soil used for nematode and soil analysis was then stored at room temperature.

Samples were classified by latitude (northern or southern coast farm location), farm of origin and cultivation method, soil texture (measured by decanting and weighting of the three soil fractions), pH (Kettler et al., 2001; Schoeneberger et al., 2012) and number of PPNs, free-living, and predatory nematodes. The soil sieving and decanting technique was used for the extraction of nematodes by suspending a 200-ml soil subsample in tap water, followed by filtering and decanting, with a set of 500- and 75-μm sieves. The filtered suspension was then examined for nematode identification and density measurement with light microscopy, using a Hawksley counting chamber at 50 ×, in three replicates. PPN were identified at the species level using available taxonomic keys (Boag and Jairajpuri, 1985; Handoo and Golden, 1989; Uzma et al., 2015; see Table 1 for variables considered) with a Leitz Orthoplan light microscope, at 312–500 ×. Hand-picked nematode specimens were placed on slides in temporary water mounts. We classified the free-living (Rhabditida and Aphelenchoides) and predatory nematodes (Mononchida and non-plant parasitic Dorylaimida) during counts using a Hawksley counting chamber at 40-100 × and available nematode descriptions (Goodey, 1963). Free-living (bacterial and fungal feeders) and predatory nematodes (mononchids and dorylaims) were counted as groups. The remaining soil was then used to measure soil texture and pH, for each sample. Spearman's correlations among nematode and soil variables were calculated using PAST (Hammer et al., 2001).

**Table 1:** Variables used for the identification of plant parasitic nematodes.

**Nematode taxa**	**Variables used**	**References/keys**
*Helicotylenchus multicinctus*	Adults tail and head shape hemispherical; presence of males; spermatheca functional and slightly offset; stylet length <24 μm; <5 lip annules; phasmids anterior to anus; lateral fields without striae; habitus = open C; V = 61–76; L = 400–673.	Boag and Jairajpuri, 1985; Uzma et al., 2015
*Pratylenchus goodeyi*	Presence of males; V = 73–75%; posterior uterine branch short, around one body width at vulva; tapering and almost pointed tail; four lip annules.	Handoo and Golden, 1989

### Metabarcoding Analyses

Rhizosphere soil samples were analyzed for the presence of bacteria and fungi using a metabacoding sequencing approach. For metabacoding analyses, 2 g of soil collected from banana roots or from controls were used from each sample. Total RNA was extracted with the RNeasy PowerSoil® Total RNA kit (Qiagen®, UK—MoBio Laboratories, Inc.), following the manufacturer's instructions. RNA concentration was determined with a Nanodrop™ spectrometer at 260 nm. The extracted material was subjected to reverse transcription according to the Illumina^TM^ sequencing protocol, using SuperScript III or IV (Invitrogen, USA), following the manufacturer's protocol. The material obtained was then purified using the QIAquick PCR Purification kit (Qiagen®, UK). The nucleic acid integrity was checked by electrophoresis on 1.5% agarose gel. The cDNA was then subjected to PCR amplification of the bacterial 16S ribosomal RNA gene and the fungal internal transcribed spacer (ITS) regions.

### 16S Data Analysis

Both ends of the V3–V4 hypervariable region were used for the amplification of the 16S rRNA ribosomal gene, which is considered capable of yielding sufficient information for taxonomic classification (Yang et al., 2002; Liu et al., 2007, 2008; Caporaso et al., 2010). The primers 341F (5′-CCTACGGGNGGCWGCAG-3′) and 805R (5′-GACTACHVGGGTATCTAATCC-3′), with affinity for flanking conserved motifs, were used to amplify the V3–V4 of the 16S hypervariable region (Van de Peer et al., 1996; Baker et al., 2003; Clarridge, 2004; Takahashi et al., 2014). MiSeq System Illumina platforms, provided by a commercial service (IGA-Technology Services, Udine, Italy^1^), were used for sequencing. Two amplification steps were used in the library workflow: an initial PCR amplification using locus-specific PCR primers and a subsequent amplification integrating the relevant flow-cell binding domains and unique indices (NexteraXT Index kit FC-131-1001/FC-131-1002), used to amplify the variable V3 and V4 regions of the 16S rRNA gene. Libraries were sequenced on a MiSeq run in paired end features with a 300-bp read length^2^.

For the bioinformatic assembly of the single read contigs, raw sequences were processed using the PandaSeq^3^ pipeline (Masella et al., 2012). Forward and reverse 16S reads were merged by applying the following PandaSeq parameters: sequences with unidentified nucleotides = filtered; lengths of overlapping region (min–max) = 100–180 nt; contig lengths (min–max) = 200–450 nt (Claesson et al., 2010). For each sample, the single fasta format file of high-quality assembled sequences was obtained by merging data, and then used as the first input for processing with QIIME 1.9 (Caporaso et al., 2010), on a Linux emulator in a Windows® 7 environment. QIIME was applied to filter the chimeras, to assemble the replicate sequences and to analyze the operational taxonomic units (OTUs) assigned through the implementation of UCLUST, applying a 97% identity threshold to discriminate at the species level (Caporaso et al., 2010). Next, an OTU table was constructed using a combined fasta file generated by *add_qiime_labels.py* using labels from a metadata mapping file, and then using *pick_de_novo_otus.py*. An OTUs.biom file was obtained by picking OTUs defined based on 97% sequence similarity, and taxonomy was assigned to individual OTUs through the Greengenes data set (ver._gg 13.5) (McDonald et al., 2012). The HDF5 OTU.biom file with sequence abundance per sample and treatments was converted to JSON.biom format using the BiomCS 1.0.6 online conversion server^4^ (ver. 1.0.6). OTUs were filtered using the sum of five sequences per OTU in total (sum of all samples) as minimum threshold, and analyzed with the graphical interface provided by Statistical Analysis of Metagenomic Profiles^5^ (STAMP, ver. 2.1.3) (Parks and Beiko, 2010; Parks et al., 2014). To compare sample pairs or samples organized into two or more groups identified by treatment and/or other traits listed in the mapping file (such as farm, crop cultivation method, soil pH or texture, density levels of phytoparasitic, free-living or predatory nematodes, sample latitude, or their combinations), the entire samples were used as the parent level with different profile levels, applying a two-tailed Student's *t*-test, with other comparative statistics. We kept unclassified OTUs and their higher levels in the analyses, by identifying the latter in the hierarchy (and eventually in STAMP plots) using the OTUs codes as tags of the higher, unclassified taxonomic levels. Heatmap plots of only significantly different OTUs [analysis of variance (ANOVA, with a 0.95 *post-hoc* Tuckey–Kramer test, filtering threshold: *p* < 0.05)] were produced with the average neighbor UPGMA algorithm and a 0.65 dendrogram clustering threshold. Two-group comparisons were performed by applying a two-sided, equal variance *t*-test (*p* < 0.05, effect size as ratio of proportions = 0.8). Principal component analysis for all samples (except the outlier sample N1) was also performed with STAMP.

The .biom OTU table was used for further analyses with the R library *mctoolsr*^6^ ver. 0.1.1.2 (R Core Team, 2013) for the production of samples Bray–Curtis dissimilarity matrices and MDS plots, for the whole data set or selected taxonomic lineages or sample groups, and for Kruskal–Wallis *t*-test and permutational multivariate analysis of variance (PERMANOVA). PAST was used to calculate sample α-diversity indices. Further R libraries used included *phyloseq* (McMurdie and Holmes, 2013), *ggplot2* (Wickham, 2016), and *boxplot* for graphics*, psych*, and *corr.test* for correlations. Venn diagrams were produced using an online public service^7^ (Heberle et al., 2015).

### ITS Data Analysis

Two amplification steps were used in the library workflow: an initial PCR amplification using locus-specific PCR primers and a subsequent amplification, integrating the relevant flow-cell binding domains and unique indices (NexteraXT Index kit FC-131-1001/FC-131-1002), used to amplify the ITS RNA gene. Libraries were sequenced in a MiSeq run in paired end with a 300-bp read length. The primers ITS1 5′-TCCGTAGGTGAACCTGCGG-3′ and ITS4 5′-TCCTCCGCTTATTGATATGC-3′ were used for the ITS locus (White et al., 1990). ITS sequences were processed by the sequencing provider using fast length adjustment of short (FLASH) reads, filtering out the sequences with unidentified nucleotides by applying 15–250 nt (min–max) lengths of the overlapping region (Magoc and Salzberg, 2011). For each sample, the single fasta format file of assembled sequences was obtained by merging data, and then used as the first input for processing with QIIME 1.9 (Caporaso et al., 2010), on a Linux emulator in a Windows® 7 environment. Next, an OTU table was constructed using *pick_otus.py* based on 97% sequence similarity and an OTUs.biom file was obtained. Taxonomy was assigned to individual OTUs using the UNITE data set (ver._7.1) using UCLUST (Edgar, 2010; Tedersoo et al., 2018). The resulting Excel® database was then edited to eliminate redundancies at the species or genus level by summing all reads counts into single representative OTUs or taxa. Unclassified OTUs were kept in the data set by adding the highest taxonomic descriptor to the unclassified, lower level tags. The same statistics and software tools applied for the 16S analysis were then used for the ITS data.

### Sequence Data Deposition

All sequence data were deposited in the sequence read archive (SRA) of the National Center for Biotechnology Information (NCBI) under accession number BioProject PRJNA540248.

## Results

### Nematodes

Plant parasitic nematodes found in the banana rhizosphere included *Pratylenchus goodeyi* Sher and Allen, and *Helicotylenchus* spp. (*H. multicinctus* Cobb, Golden, and *H. abunaamai* Siddiqi), found in 45% (density range: 67–1,750 × 100 cc soil^−1^) and 70% (60–2,300 × 100 cc soil^−1^) of samples, respectively. *Pratylenchus goodeyi* is a severe root endoparasite of banana worldwide, with a migratory phase in the soil on or around the roots. This species is considered to have been introduced to the Canary Islands, likely through the infested plant propagation material. *Helicotylenchus multicinctus*, a further severe banana ecto–endoparasite, was more prevalent in banana samples. In control samples, *P. goodeyi* was not detected, whereas *Helicotylenchus* spp. were only found in one sample (Supplementary Table 2).

Free-living nematodes included Rhabditidae as bacterial feeders and fungal feeders such as *Aphelenchoides* spp. Microbial feeders were present in 79% of the samples (density range: 120–13,533 nematodes × 100 cc soil^−1^) (Supplementary Table 2). Predatory nematodes belonged to Dorylaimida and Mononchida. They were found in 26% of the samples (60–360 nematodes × 100 cc soil^−1^) (Supplementary Table 2).

Soil profiles showed most prevalent texture class as sandy clay, with a mean pH for all samples around 7.4 (min-max: 6.3–8.8) (Supplementary Table 2).

Spearman's correlations of *P. goodeyi* and *Helicotylenchus* densities with soil variables showed a significant inverse relationship with sand and a positive correlation with the other soil fractions. A significant positive correlation also occurred between the density of *P. goodeyi* and the number of *Helicotylenchus* spp., as well as between the latter and the density of free-living nematodes. Predatory nematodes were only negatively correlated with soil clay content (Table 2).

**Table 2:** Spearman's rank correlation coefficient (ρ) among nematode population densities and other soil variables^*^.

	***Pratylenchus goodeyi***	**Predatory nematodes**	***Helicotylenchus* spp**.	**Free living**	**pH**	**Sand (%)**	**Silt (%)**	**Clay (%)**
*Pratylenchus goodeyi*		*0.59213*	***0.00116***	*0.14411*	*0.55280*	***0.00031***	***0.00735***	***0.00026***
Predatory nematodes	−0.08973		*0.10610*	*0.93726*	*0.30108*	*0.24468*	*0.73659*	***0.00932***
*Helicotylenchus* spp.	**0.50705**	−0.26628		***0.02984***	*0.18466*	***0.00000***	***0.00364***	***0.00001***
Free living	0.24150	0.01321	**0.35276**		*0.34169*	***0.00077***	***0.00059***	*0.08825*
pH	−0.09937	−0.17225	0.21990	0.15856		*0.47186*	*0.97493*	*0.39637*
Sand (%)	–**0.55431**	0.19339	–**0.67035**	–**0.52229**	−0.12030		***0.00000***	***0.00000***
Loam (%)	**0.42803**	–**0.05641**	**0.46034**	**0.53208**	−0.00527	–**0.87657**		***0.00055***
Clay (%)	**0.55959**	–**0.41634**	**0.66588**	0.28033	0.14162	–**0.81552**	**0.53408**	

* *All samples (n = 37). Upper matrix shows p levels (italics, significant values at p < 0.05 are shown in bold). Lower matrix shows ρ values (significant coefficients are shown in bold)*.

### 16S Data

A total of 4,426,081 single reads of the 16S V3-V4 region were obtained from the 37 samples analyzed (one sample was discarded due to a low number of reads). PandaSeq produced 1,250,383 contigs that were analyzed with QIIME, yielding a total 469,205 sequences, used for taxonomic assignments in each sample. The 3,938 OTUs obtained after filtering were represented among the different samples with different frequencies, of which only 68 (1.7%) were classified at the species level. The OTUs belonged to 190 classified genera (2,667 OTUs unclassified at this level), 128 families (1,166 unclassified), 400 orders (308 unclassified), 97 classes (31 unclassified), and 21 bacterial phyla. Archaea were represented by only 14 OTUs from phyla Crenarcheota and Euryarchaeota.

**Figure 1:**
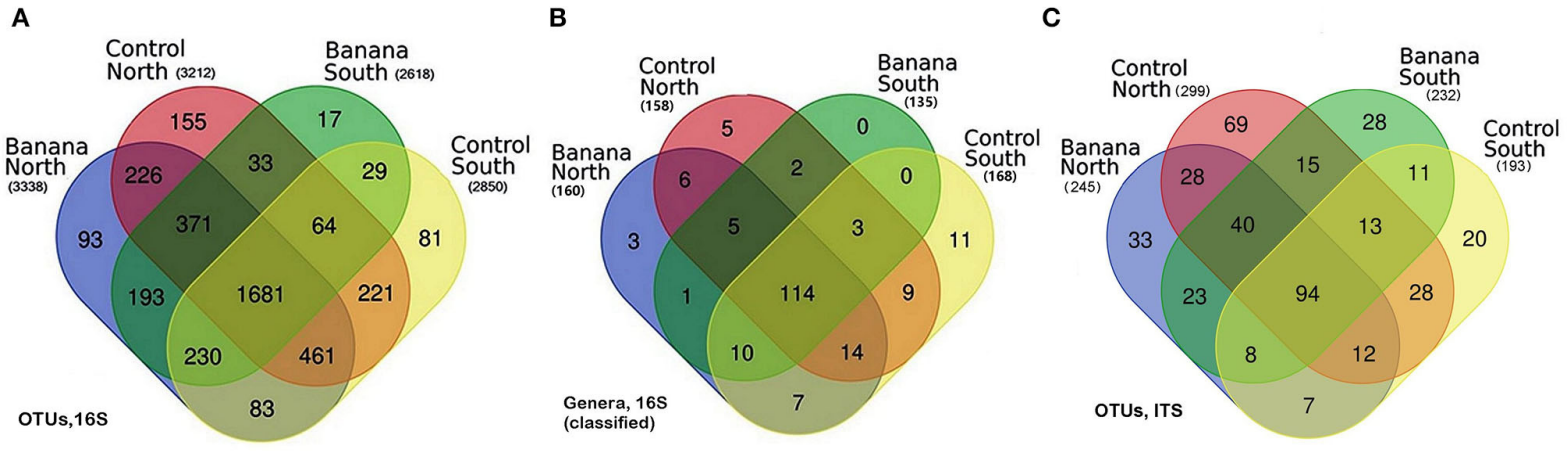
Venn diagrams showing the distribution of bacterial operational taxonomic units (OTUs) **(A)**, classified genera **(B)**, and fungal ITS OTUs **(C)**, among all samples, grouped by crop and latitude.

Venn diagrams showed a core microbiota of 1,681 OTUs, either classified or not, from 190 classified genera, of which 114 were in common for all samples. A higher number of OTUs were observed in northern samples, which also showed the highest number of unique OTUs (Figures 1A,B).

**Figure 2:**
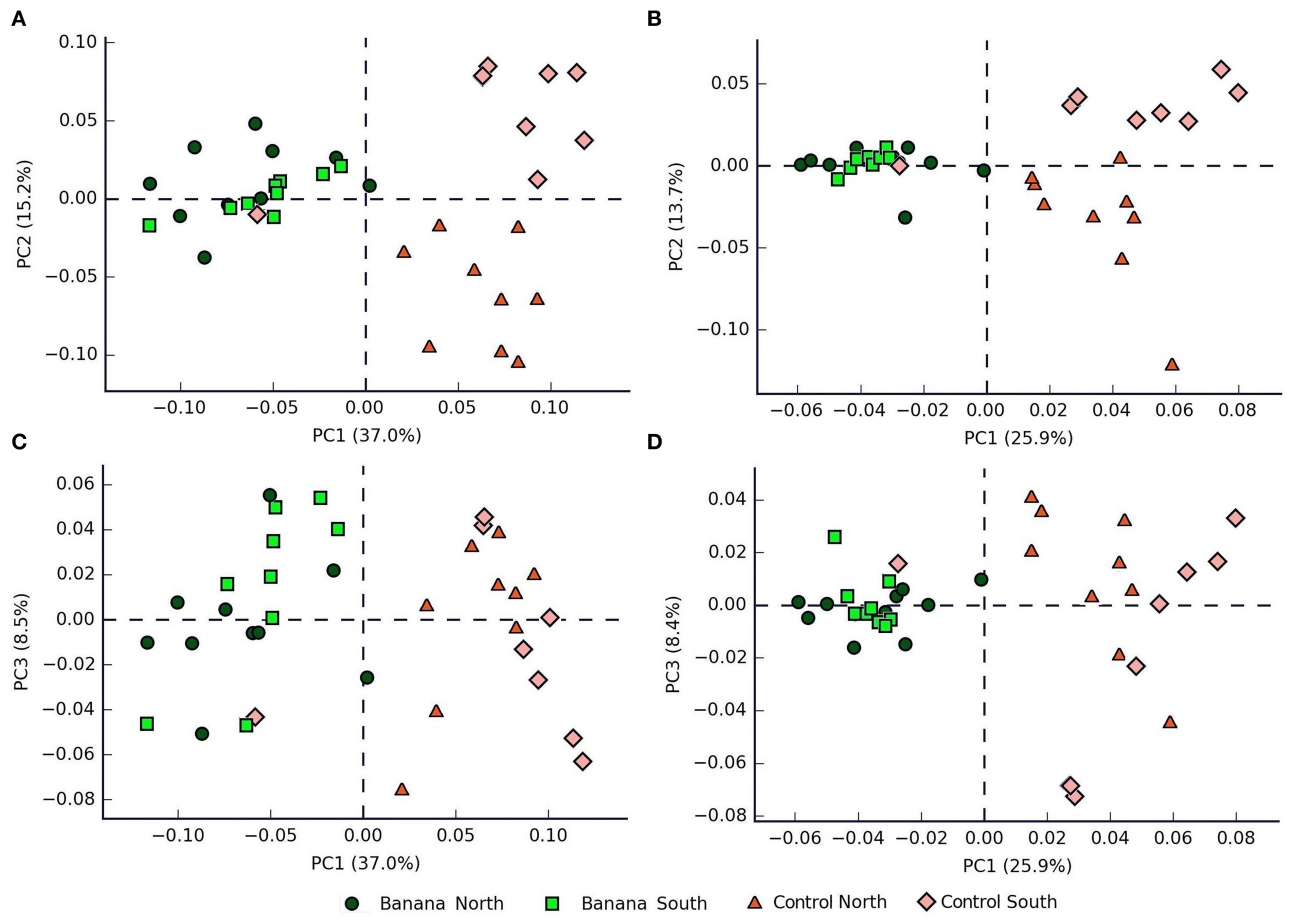
Principal component analysis (PCA) plots (**A,B** = PC1 vs. PC2, **C,D** = PC1 vs. PC3) showing samples classified by crop and latitude, based on 16S sequence data, at the family **(A,C)** and genus **(B,D)** levels (all samples except N1).

**Figure 3:**
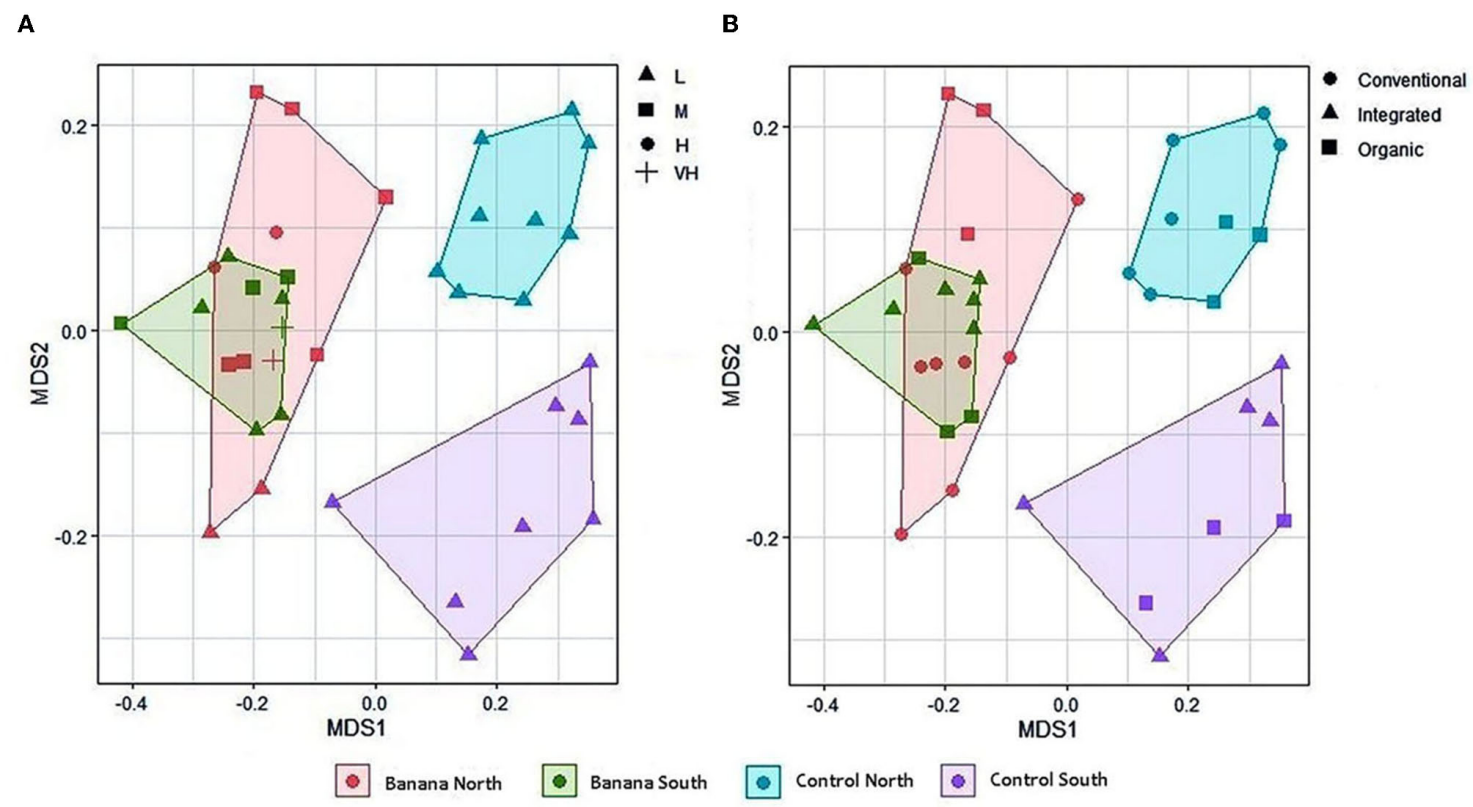
Multidimensional scaling (MDS, stress = 0.14) plots of all samples based on latitude and density of *Helicotylenchus* spp. **(A)**, and crop cultivation method **(B)**. Nematode density levels (expressed as adult and juvenile nematodes × 100 cc soil^−1^) are: L = low or absent (0–290), M = medium (291–804), H = high (805–1,319), VH = very high (> 1,319) (mean density and standard deviation (SD) = 290 ± 514 nematodes × 100 cc soil^−1^).

Principal component (PCA) plots showed distinct separations among the sample groups (Figure 2A) in relation to latitude and presence of banana roots, as did non-metric multidimensional scaling (NMDS) analyses. The clusterings reflected changes in the microbiota, with groups accounting for the presence and density level of *H. multicinctus* and for the effect of the crop cultivation method and latitude (Figures 3A,B). A more homogeneous clustering was observed in the PCA plots at the genus level, indicative of a higher similarity among the samples, at a lower taxonomic level (Figure 2B). PERMANOVA analysis showed significant differences in false discovery rate (FDR) among groups, mainly related to the presence/absence of banana roots, latitude, and number of *Helicotylenchus* spp. (Supplementary Table 3).

**Figure 4:**
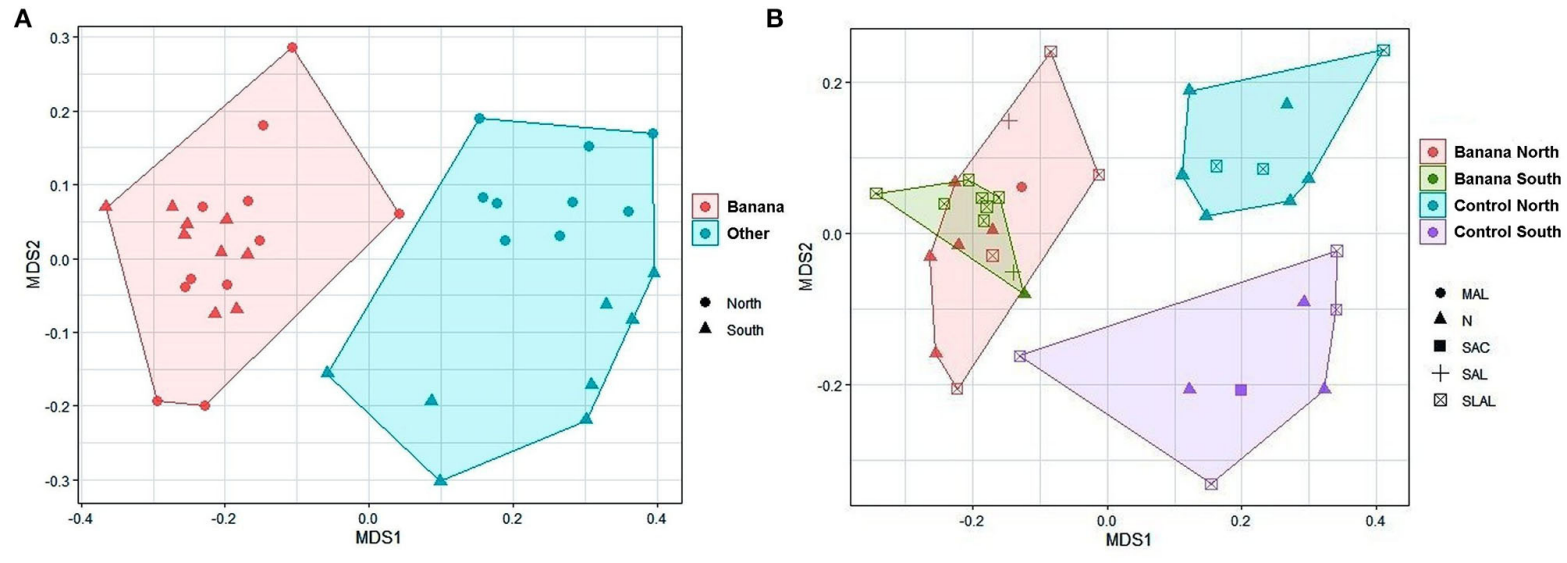
MDS plots (stress = 0.14) of all samples when considering only Proteobacteria (rarefied to 1,800 taxa) and based on latitude and crop **(A)**. MDS plots of only Rhizobiales (**B**, rarefied to 940 taxa, stress = 0.14) with crop, latitude and soil pH. MAL, moderately alcaline; N, neutral; SAC, slightly acid; SLAL, slightly alcaline; SAL, strongly alcaline.

Separated sample clusterings were also shown by NMDS when the analysis was limited to specific taxa, such as Proteobacteria, separating samples by crop and latitude (Figure 4A), or Rhizobiales, with clusters distinguished by latitude and soil pH (Figure 4B).

**Figure 5:**
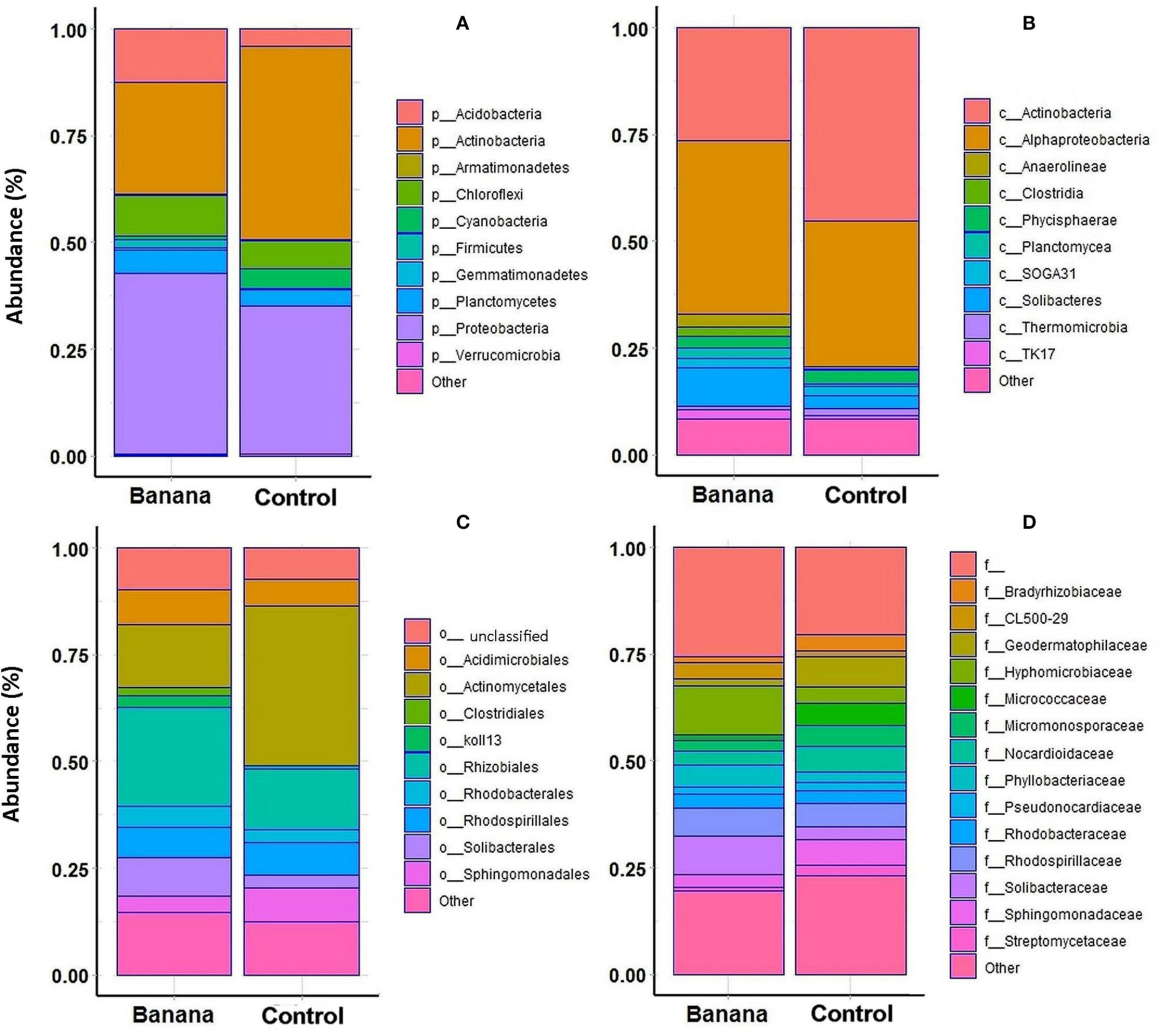
Top 10 more represented phyla **(A)**, classes **(B)**, orders **(C)**, and 15 more represented families **(D)**, in the samples proceeding from *Musa acuminata* rhizosphere vs. adjacent banana root-free control sites.

**Figure 6:**
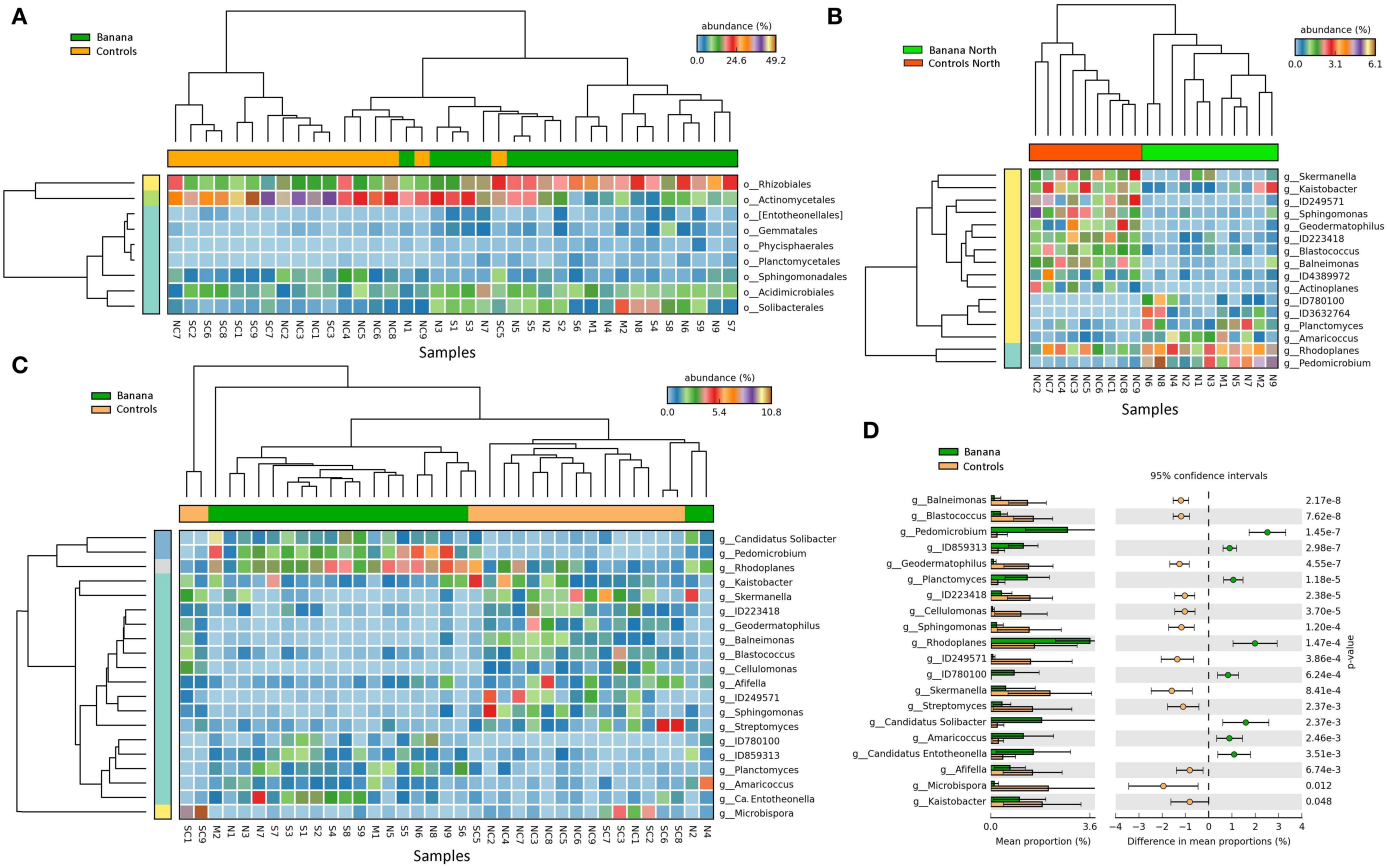
Heatmaps of 16S rRNA sequence abundance among all samples **(A,C)**, and in a subset of northern samples **(B)**, showing the separation of taxa between banana plant rhizosphere and adjacent controls, at the order **(A)** and genus **(B,C)** levels (filtered at *p* < 0.05, effect size filter for difference or proportions = 0.8). Extended error bar plot showing the mean sequence proportions for differentially represented bacterial taxa (*p* < 0.05), at the genus level **(D)**.

A higher representation of Proteobacteria, Acidobacteria, and Chloroflexi was observed in the banana rhizosphere, when considering the most abundant taxa by comparing plant and control samples, whereas Actinobacteria were more prevalent in adjacent control sites (Figure 5A). This repartition was reflected in a higher prevalence of the classes Alphaproteobacteria, Clostridia, Solibacteres, and Anaerolineae in the banana rhizosphere, with Actinobacteria more represented in control sites (Figure 5B). At the order level, a higher frequency of Rhizobiales and Solibacterales characterized the banana rhizosphere samples, with Actinomycetales more prevalent in the control soils (Figures 5C, 6A). The families Hyphomicrobiaceae and Solibacteraceae were more prevalent in the banana rhizosphere samples, whereas members of Bradyrhizobiaceae were more represented in controls (Figure 5D). A similar distribution was observed at the family level when samples were grouped by crop and latitude (Table 3). Differences between banana and control samples were also observed at the genus level, with distinct clusterings (Figure 6C), more evident when considering only the northern samples (Figure 6B). The data showed a higher abundance of *Pedomicrobium, Rhodoplanes* with other unclassified taxa in banana rhizosphere, whereas *Microbispora, Kaistobacter, Ca*. Solibacter, and *Skermanella* were more represented among the controls (Figure 6D).

**Table 3:** Most represented bacterial families and relative abundance as shown by 16S sequence reads, accounting for differences among banana rhizosphere, and other adjacent, crop-free control sites. Samples were classified by crop and latitude.

	Abundance (%)*						
**Families**	**Banana North**	**Banana South**	**Control North**	**Control South**	***P***	Bonferroni P**	**FDR P**
Geodermatophilaceae	0.013	0.018	**0.074**	0.062	2.470368e-05	**0.00027**	**0.00027**
Bradyrhizobiaceae	0.011	0.014	**0.052**	0.023	1.058206e-04	**0.00116**	**0.00058**
Solibacteraceae	**0.092**	0.089	0.035	0.022	1.656480e-04	**0.00182**	**0.00060**
Hyphomicrobiaceae	0.114	**0.116**	0.039	0.036	1.671030e-04	**0.00183**	**0.00045**
Phyllobacteriaceae	**0.056**	0.048	0.015	0.030	2.181649e-04	**0.00239**	**0.00047**
Sphingomonadaceae	0.026	0.031	**0.075**	0.044	5.658774e-04	**0.00622**	**0.00103**
Micromonosporaceae	0.017	0.029	**0.079**	0.018	6.404280e-03	0.07044	**0.01006**
Nocardioidaceae	0.033	0.032	0.045	**0.076**	8.032507e-03	0.08835	**0.01104**

*Min. 5%, in at least one sample group. Most represented families are shown in bold (analyzed with the R library mctoolsr).

** *p-values based on Kruskal–Wallis tests, with Bonferroni and false discovery rate (FDR) corrections (significance at p < 0.05 is shown in bold; rarefaction level applied = 4,500 sequences per sample; total samples retained = 37)*.

### ITS Data

A total of 1,725,692 contigs were obtained from 37 samples analyzed with QIIME, yielding 572,389 ITS sequences. After filtering out sequences from plants and other clades, taxonomic assignments for the Kingdom Fungi across all samples yielded a total of 429 OTUs classified into six phyla and 24 classes. The classification showed fungi in 60 orders (with 15 additional ones unclassified), 106 families (with 48 additional unclassified), and a total of 314 genera (65 of which were unclassified). OTUs classified to the species level were 189, whereas 175 could be only assigned at the genus level, with 65 OTUs also unclassified at the species level.

Venn diagrams showed a common core fungal microbiota of 94 OTUs, either classified or not, including BCA such as *Metarhizium anisopliae* (NCBI accession n. JN377427), *Arthrobotrys oligospora*, and *Acrostalagmus luteoalbus* (AJ292420). A higher frequency of OTUs was observed in the northern control and banana samples, which also showed the highest number of unique OTUs (Figure 1C). Several fungal species known as predatory or parasitic on nematodes or other fungi showed, in the banana rhizosphere samples, higher frequencies and/or sequence numbers, including *P. chlamydosporia, M*. *anisopliae*, and species of genera *Arthrobotrys, Beauveria, Dactylaria, Dactylella, Lecanicillium, Nematoctonus*, and *Trichoderma* (Supplementary Tables 4, 5). OTUs also included plant pathogenic fungi such as *Musicillium theobromae* (EF543859; JQ647444), the causal agent of cigar end rot (found in northern and southern banana samples), *Macrophomina phaseolina* (KF766195) (core microbiota), or other pathogens belonging to the genera *Alternaria, Cladosporium*, and *Fusarium*. Arbuscular mycorrhizal fungi (AMF) included *Funneliformis* spp. (HF970250) and the ericoid mycorrhiza *Oidiodendron* spp. (KF156313, AF062793 core microbiota). Species reported in the literature as human pathogens, i.e., *Basidiobolus ranarum, Lichtheimia corymbifera* (GQ342878), or *Actinomucor elegans*, were also recorded (JN205828) (Supplementary Table 5).

**Figure 7:**
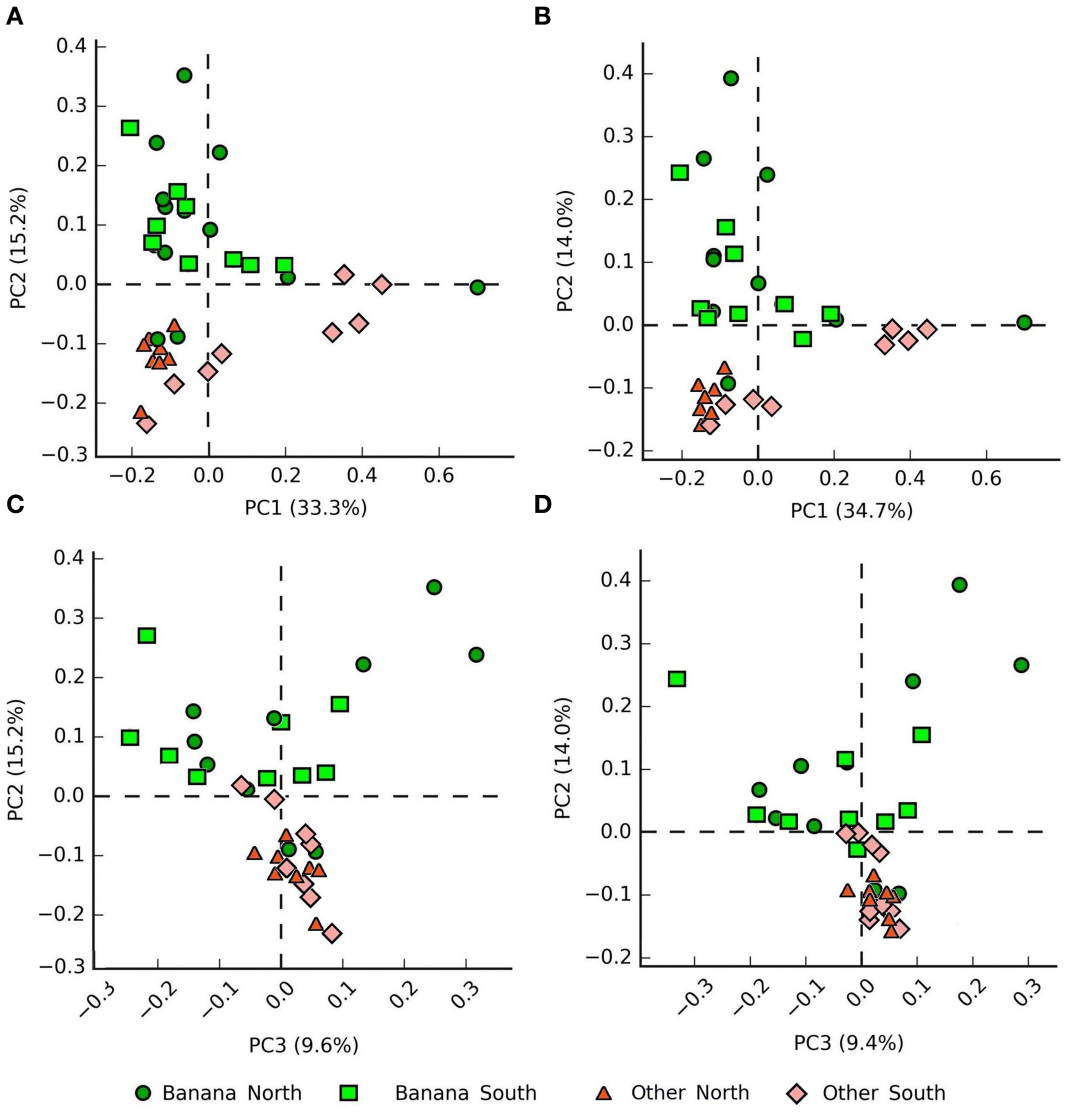
PCA plots (PC1 vs. PC2 and PC1 vs. PC3) by crop and latitude of the samples based on internal transcribed spacer (ITS) data, showing clustering at the family **(A,C)** and genus **(B,D)** levels.

Internal transcribed spacer PCA plots showed a clear repartition of samples in relation to the presence/absence of banana plants and latitude, visible at different taxonomic levels (Figures 7A,B).

**Figure 8:**
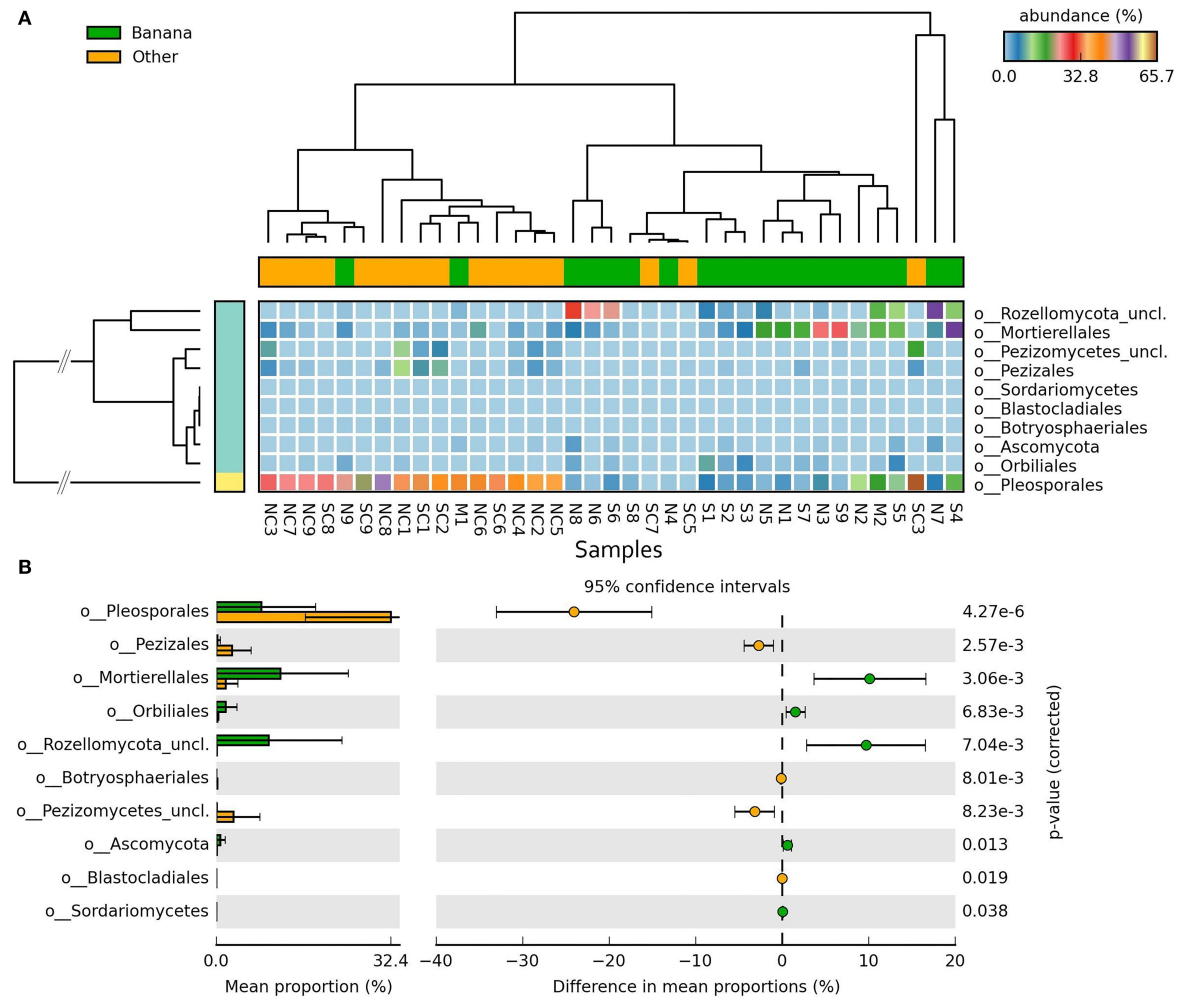
Heatmaps of ITS sequence abundance among all samples showing the differential groupings of fungi between banana plants rhizosphere and adjacent controls, at the order level **(A)**, shown by hierarchical cluster analysis (filtered at *p* < 0.05, effect size filter for difference or proportions = 0.8). Error bar plots of differences in mean proportions among samples **(B)**.

**Figure 9:**
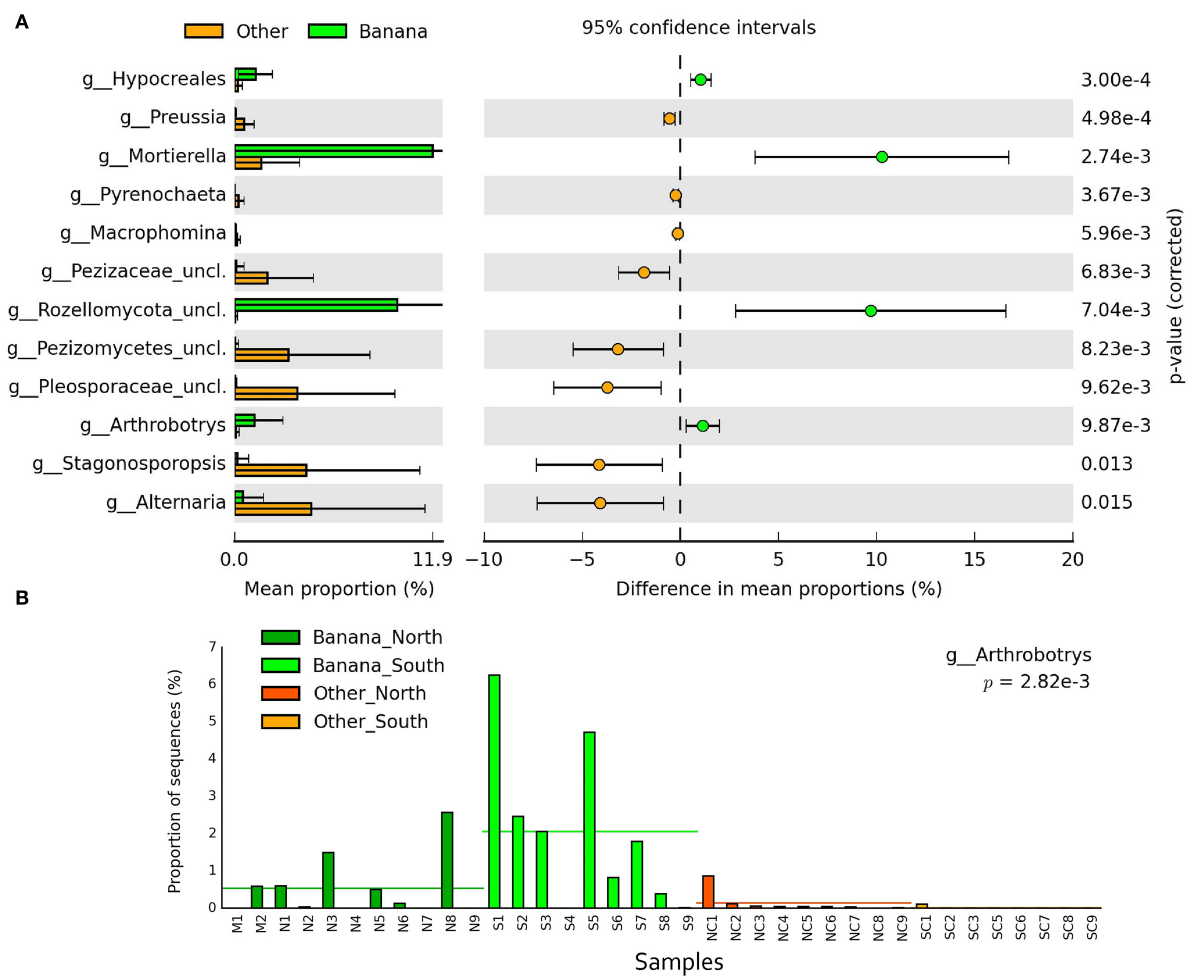
Error bar plots (*p* < 0.05) showing the differences of ITS sequence abundance (mean proportions) among samples at the genus level **(A)**. Bar plot showing the proportion of sequences for the nematophagous genus *Arthrobotrys*, considering rhizosphere and control samples, and latitude **(B)**.

Differences in taxa representation between the banana and control sample groups were observed for mycoparasitic fungi from the phyla Rozellomycota (endoparasites of other fungi) and Zygomycota (i.e., *Mortierella* spp.), which were more represented in the banana rhizosphere, whereas Ascomycota and Basidiomycota were more frequent among adjacent controls (Figure 8A; Supplementary Figures 2A,B). Hierarchical cluster analysis of samples using ITS sequence abundance showed a major effect of banana plants and, to a lesser extent, of latitude, whereas the crop cultivation method did not appear to affect the clusterings (Supplementary Figure 2C). Species of nematophagous fungi within Orbiliales appeared more represented in the banana samples, although with a low proportion, mostly represented by *Arthrobotrys* (Figures 8B, 9A,B; Supplementary Table 4), together with Mortierellales and an unclassified order from the phylum Rozellomycota (Supplementary Figure 3).

The more represented fungi at the family or genus level were analyzed by comparing sequence representation (%) in samples grouped by crop and latitude, as well as by other classification variables. Differences were found for fungal families in relation to the sample origin (banana rhizosphere vs. controls), age of crops, density levels of predatory or free-living nematodes, plant germplasm, and location of farms (Supplementary Figure 4). When comparing banana rhizosphere vs. control samples, the most represented families showed a higher abundance of Microascaceae, Wallemiaceae, and Hypocreaceae in the banana rhizosphere, whereas AMF (Glomeraceae and Claroideoglomeraceae) were more abundant in the control soils, as also in younger (<5 years old) plantations (Supplementary Figures 4A,B). Considering the location, an effect was shown for fungi associated with organic matter (with a higher abundance of Wallemiaceae in the northern samples, and Trichocomaceae in the southern ones) together with a higher abundance of Glomeraceae and Hypocreaceae in the northern farms (Supplementary Figure 4E).

Considering both combined factors, a higher representation was found for *Wallemia* spp. (most represented in the banana northern fields >40 years old), with Hypocreaceae, a *Petriella* sp. and an unclassified member of Claroideoglomeraceae (most frequent in the northern and southern banana samples). Unclassified members of Glomeraceae and Bolbitiaceae were more represented in the northern controls, whereas *Metarhizium* spp. with an unclassified Cystolepiota were most common in the southern controls. An unclassified member of Hypocreaceae was more represented in conventional crops when grouping samples by the cultivation method, whereas *Arthrobotrys* and *Microascus* spp. were more frequent in integrated crops (Supplementary Table 6). Banana cv Gruesa showed, when compared to the most prevalent cv Gran Enana and controls, a low abundance of Claroideoglomeraceae and Microascaceae, with a higher content of Vibrisseaceae and an unclassified family within Pezizomycetes (ectomycorrhizae) (Supplementary Figure 4D).

Grouping by soil type showed a significantly higher representation of: *Penicillium, Coniochaeta*, and an uncl. Chaetomiaceae in clay soils, *Leucoagaricus* with an uncl. Agaricales in sandy soils, *Phialocephala* and *Wallemia* in sandy-clay soils, *Sagenomella* in sandy-clay-loamy soils, and *Metarhizium* in sandy-loamy soils. For the effect of pH, *Diaporthe* was more frequent in moderately alkaline soil (pH 7.9–8.4), and an unclassified member of Zygomycota was more represented in slightly acidic soil (pH 6.3–6.5) (Supplementary Table 6).

Grouping by nematodes showed a higher representation of *Wallemia, Petriella, Phialocephala*, and *Ceratobasidium* with higher densities of *Helicotylenchus* spp., and of *Diaporthe* and *Petriella* with medium densities of *P. goodeyi* (Supplementary Table 6). *Phialocephala, Paraconiothyrium, Coniothyrium, Meyerozyma*, and *Beauveria*, with unclassified members of Glomerales, Bionectriaceae, and Sordariomycetes, were significantly more represented in samples with high densities of free-living nematodes. An unclassified member of Tulasnellaceae, along with *Zopfiella* spp. and *Cunninghamella* spp., were more represented in samples with medium to highest densities of predatory nematodes (Supplementary Table 6). Higher densities of predatory nematodes were associated with a higher abundance of Wallemiaceae and Hypocreaceae, whereas free-living nematodes were associated with an increase in Debaryomycetaceae (budding yeasts), Vibrisseaceae, and an unclassified family within Saccharomycetales (Supplementary Figures 4C,F).

Plant pathogenic fungal genera *Alternaria, Macrophomina*, and *Pyrenochaeta* along with others were mostly found in control samples, except *Ceratobasidium* and *Chaetomium*, which were more represented in the banana rhizosphere samples (Supplementary Figure 5).

A number of diversity indexes were calculated for the ITS data to verify the effects of cropping and latitude on the sample groups examined. Dominance levels in all sample groups were low (0.1–0.2 range), indicating that there were no outstanding taxa (Supplementary Tables 7A,B). As shown by the mean comparisons (Student's *t*-test, *p* < 0.05, Supplementary Table 7C), the α-diversity analysis for the ITS data showed no significant differences between banana samples when they were only grouped by southern vs. northern latitude. On the contrary, most indexes showed significant differences when adjacent, northern, and southern controls were compared. Comparison of northern banana plants vs. adjacent controls showed an effect on the number of taxa, richness (Menhinick, Margalef, and Fisher alpha), and Chao-1 only, indicating that the effect occurred at the level of the rarest taxa. The northern controls also differed significantly from the southern banana and control groups, for the same indexes. Comparison of the southern banana samples vs. adjacent controls showed differences for individuals, Simpson and Shannon indexes, and Menhinick and Margaleff indexes, confirming differences for species richness, as for all sample groups (Supplementary Table 7C).

### Cooccurrence and Correlations

The correlations between nematodes and fungi or bacteria appeared specific to each nematode species or functional group, when density data and the number of sequences were used. Spearman's correlations showed positive links of *P. goodeyi* densities with bacteria from the genera *Pedomicrobium, Afifella, Pilimelia, Hyphomicrobium, Rhodoplanes, Microlunatus, Clostridium*, and *Streptomyces*, and unclassified members of Solibacterales and Rhizobiales, Rhodospirillaceae and Ellin329. Significant inverse relationship were found for this nematode with an unclassified member of Rhodobacteraceae and, among fungi, with *Zopfiella* (Sordariomycetes), *Ceratobasidium* (Cantharellales), unclassified Pezizomycetes, *Spiromastix, Sordaria, Arthrographis*, and *Chaetomium* (Supplementary Table 8).

A broader set of taxa was correlated with *Helicotylenchus* spp. Positive correlations included, among fungi, the plant pathogen *Thanatephorus cucumeris* (teleomorph of *Rhizoctonia solani*), *Mortierella oligospora, Cyphellophora, Sarocladium*, and unclassified Rozellomycota, Hypocreales, Stramenopiles, Eurotiales, and Onygenales. Among bacteria, positive correlations included members of the genera *Iamia, Amaricoccus, Planctomyces*, and *Kribbella*. Unclassified taxa positively correlated with *Helicotylenchus* spp. included members of different bacterial lineages, both unclassified (C111, X0319_7L14, S085, WD2101, SAR202, Ellin6529, Ellin6075, and Ellin329) or classified (Nocardioidaceae, Sphingomonadales, Micrococcales, Solibacterales, Hyphomicrobiaceae, Hyphomonadaceae, Erythrobacteraceae, Pseudonocardiaceae, and Acidimicrobiales) (Supplementary Table 8). As for *P. goodeyi*, inverse correlations were also found for *Helicotylenchus* spp. with *Zopfiella* and, among the fungi, *Chaetomium and Spiromastix*. Further negative correlations for these nematodes included members of Acidimicrobiales, Rhodobacteraceae, and the fungus *A. luteoalbus*.

Fungi correlated with predatory nematodes (dorylaims and rhabdites) included *Venturia* spp., *A. luteoalbus*, an unclassified member of Mytilinidiaceae and a nematophagous *Dactylella* spp. Negative correlations involved the fungus *Malassezia globosa*, and unclassified bacterial taxa from *Streptomyces*, Caulobacteraceae, Rhodospirillales, and Acidimicrobiales (Supplementary Table 8).

Free-living nematodes were positively correlated with a different group of bacteria, including *Cryptococcus randhawii* and members of the genera *Rhodoplanes, Roseomonas, Sphingomonas, Leptolyngbya, Kaistobacter, Streptomyces*, and *Microlunatus*, with further unclassified taxa (members of Caldilineaceae, *WD2101*, Ellin6075, Acidimicrobiales, Bradyrhizobiaceae, and Solibacterales). Fungi positively correlated with free-living nematodes included *Stemphylium herbarum*, species from the genera *Articulospora, Westerdykella, Stramenopiles, Cyphellophora, Mortierella*, and members of Olpidiales, Chaetothyriales, and Agaricales. Inverse correlations involved *Streptomyces* spp., *Mesorhizobium* spp., and members of Nocardioidaceae, Rhodospirillaceae, Rhodobacteraceae, S085, and Ellin6529. Among fungi, negative correlations were found with *Spiromastix* sp. and a member of Sordariomycetes (Supplementary Table 8).

*Arthrobotrys* and *Microascus* were significantly more represented on farms applying IPM, whereas an unclassified member of Hypocreaceae was more represented on conventional farms (Supplementary Table 6). A plant pathogenic fungus, *Diaporthae* spp., and an organic matter decomposer, *Petriella* spp., were more represented at medium densities of *P. goodeyi* (151–533 nematodes × 100 cc soil^−1^). The latter was also more represented, with *Wallemia* and the endophytes *Phialocephala* and *Ceratobasidium* spp., an orchid mycorrhyza and biocontrol agent, respectively (Mosquera-Espinosa et al., 2013), in samples with higher *Helicotylenchus* spp. numbers (Supplementary Table 6).

Positive or negative correlations with soil pH were found within the same bacterial lineages (i.e., Acidimicrobiales and Rhodospirillaceae). Taxa most negatively correlated with pH included unclassified members of Ellin329, AKIW874, S085, Alphaproteobacteria, Rhodospirillaceae, Hyphomonadaceae, Rhodospirillaceae, with *Ca*. Solibacter, *Pilimelia* spp. and, among fungi, *Mortierella capitata* (Supplementary Table 8).

## Discussion

Metabarcoding data showed a clear separation of microbial and fungal samples on the PCA and MDS planes, with clusterings associated with the presence/absence of banana roots, and latitude (Figures 2–4, 6). The representation of the samples hence appears indicative of both anthropogenic effects due to cultivation and, to a lesser extent, farm local climate, which affects a number of microbial taxa. The local climate depends on latitude, differing between northern areas (more humid and cooler, due to exposure to trade winds and ocean currents) and southern zones (arid and more exposed to Sahara heatwaves).

Both climate and aboveground cover have been consistently recognized as key factors influencing soil microbiota profiles. The effect of the aboveground cover on belowground microorganisms has been reported under different agroecological conditions, ranging from pot trials to natural ecosystems. Cropping affects soil microorganisms through many factors including cultivation regimes, plant cover and age, root exudates, and soil types (Berg and Smalla, 2009; Reinhold-Hurek et al., 2015; Compant et al., 2019; Delitte et al., 2021). Experimental data from assays carried out under controlled conditions (with faba bean and wheat plants) for example, showed an effect of the plant species on the soil fungal communities profiles, with differences related to the sample origin (bulk soil or rhizosphere). The tested cropping regimes (monocultures vs. intercroppings) also showed an effect of rhizosphere soil on bacterial diversity and richness (Granzow et al., 2017).

The differences observed between the banana and control samples were also reflected in the different fungal families (Wallemiaceae, Microascaceae, and Agaricaceae) and genera (unclassified member of Claroideoglomeraceae), which were more represented in the banana samples (Supplementary Table 6). These changes, including organic matter decomposers and an AMF, appear to be consistent with the introduction and cultivation of the plants. The mechanisms underpinning such an effect are likely related to the farming practices applied, including an enriched organic matter content in the first soil layers, due to the practice of covering the soil surface with banana decaying leaves, adopted locally by farmers. Fertilization, organic matter decomposition, root exudates, and irrigation are likely factors responsible for the observed changes in microbiota structures. Metabarcoding data on banana root endophytes, proceeding from the same environment, showed significant differences between mother plants and suckers, with *Pseudomonas* spp. as the most prevalent endophytic bacteria (Gómez-Lama Cabanás et al., 2021). The microbial community structure originally present in the soil is hence dependent on microenvironmental changes, shifting composition profiles from soil to rhizosphere, up to plants tissues, suggesting a progressive adaptation to the changes that characterize the colonized microhabitats.

The metabarcoding data also showed the presence of several known BCA species of nematodes or fungi. Microbial antagonists of nematodes have a potential to naturally regulate or even suppress PPN numbers, as well as to induce a defense reaction in plants (Oka, 2010; Liang et al., 2019; Topalović and Heuer, 2019). Although correlations may not be considered as a sufficient determinant of an antagonistic or cooperative/exclusion interaction, our data showed specific links of taxa with the variables considered. However, only a limited number of known nematophagous species was directly linked to PPN in the banana samples. Surprisingly, the co-occurrence analysis showed that, apart from the nematode trapping fungus *A. oligospora*, the bacterial and fungal taxa correlated to nematode densities did not include known nematophagous BCA species (Supplementary Table 8). The data instead indicated an effect of the nematode guilds as each considered group (two plant parasites, a free-living and a predatory species group) showed significant correlations with a specific subset of unique bacteria and fungi. In particular, the densities of both *P. goodey* and *Helicotylenchus* spp. showed positive correlations with different microbial species. The latter was correlated with a broader range of taxa, different from that of *P. goodeyi*, including, among others, the plant pathogenic fungus *T. cucumeris* (teleomorph of *R. solani*), with other unclassified and poorly studied bacterial lineages (i.e., C111, WD2101, SAR202, and Ellin6529). Moreover, a few significant negative correlations with fungi or bacteria were found for both PPN groups, that included *Spiromastix* spp., a genus of fungi producing antibacterial compounds (Niu et al., 2014), also inversely correlated to free-living nematodes (Supplementary Table 8). Negative correlations were found more frequently for free-living, microbiovorous nematodes, likely related to the short life cycle of microbiovorous species, to their direct exposure to fungi or bacteria and possibly to the release of microbial toxic compounds. In this case, a microbial antagonistic activity affects hosts and antagonists almost at the same time, yielding inverse relationships. As the PPN spend part of their cycle in the roots a delay usually occurs between the pathogen and nematode density shifts, affecting correlations. This may be also due to the PPN longer life cycle (4–5 weeks), and to the more complex dispersal and infection strategies of their antagonists.

Data thus indicate that the links between the nematophagous BCA species found and their target PPN hosts are more complex than expected, characterized also by a microbial trophic range and adaptive metabolic capability likely wider than known. An example of this complexity is given by OTUs classified in the genus *Beauveria*, a fungus usually associated with insects, whose higher frequency in banana samples and higher representation with the highest densities of free-living nematodes (together with other BCA of fungi such as *Paraconiothyrium*) (Supplementary Tables 4, 6) is worth further investigation. Despite suggestive of an association, direct assays are needed to establish a dependence of *Beauveria* spp. on nematodes, as both may be correlated to a further, unknown factor (i.e., increased invertebrate densities in the rhizosphere due to higher fertility levels or enhanced freeder roots development). Further assays are hence needed to clarify this aspect. Few data are, moreover, available in the literature for a *Beauveria* nematophagous activity, apart from an isolate of *Beauveria bassiana* obtained from cysts of *Heterodera filipjevi* and successfully tested as a BCA vs. the nematode juvenile stages, in controlled conditions (Zhang et al., 2020).

The data also suggest a probable association of the entomopathogenic fungus *M. anisopliae* with insects other than the banana weevil *C. sordidus*, which may represent a secondary host, as the fungus was more represented, with similar frequency levels, in the control samples. An inverse relationship of this fungus with soil pH was also found (Supplementary Table 4). A phylogenetically close species, the endophyte *P. chlamydosporia*, was also found in the banana rhizosphere samples, although with a low frequency (Supplementary Table 4). As an endophyte, this species is capable to colonize banana roots, inducing plant growth promotion (Mingot-Ureta et al., 2020). The fungus is also a nematode egg parasite, and is known to activate defense-related genes in endophytically colonized roots (Manzanilla-López et al., 2013), an effect also observed in banana aboveground tissues (Tolba et al., 2021). This fungus may also produce secondary metabolites with insecticidal activities (Lacatena et al., 2019).

The nematode BCA data also showed a number of *Trichoderma* spp., which were found with a higher frequency in the banana samples (Supplementary Table 4). Several *Trichoderma* spp. have been reported as an efficient BCA of nematodes, including *Trichoderma harzianum* (Sharon et al., 2001; Sahebani and Hadavi, 2008; Fan et al., 2020). However, no correlation was found for *Trichoderma* spp. with PPN among the samples examined (Supplementary Table 8). Moreover, an effective role of *Trichoderma* spp. against plant pathogens, i.e., *Fusarium* spp., has been reported for many hosts, including *Musa* spp. (Sivan and Chet, 1986; Thangavelu and Gopi, 2015; Chaves et al., 2016; Bunbury-Blanchette and Walker, 2019).

The densities of *P. goodeyi* and *Helicotylenchus* spp. in soil varied largely among samples. The endoparasite *P. goodeyi* was only found in the northern banana fields, with a maximum density of 1,100 nematodes × 100 cc soil^−1^. *Helicotylenchus* spp. were found in 90% of the northern samples and <10% in the southern samples, with a maximum of 2,300 individuals × 100 cc soil^−1^ (Supplementary Table 2; Supplementary Figure 1). Both nematodes are severe parasites of *Musa* spp., and may reach high densities in roots (Ssango et al., 2004; Roderick et al., 2012). Due to the low amounts of roots collected and required for metabarcoding analyses, nematode densities were preferably assessed in the soil. Density levels appeared, however, sufficient to sustain a severe root infestation, and compatible with the field data reported on other *Musa cvs* from other regions (Talwana et al., 2000; Aguirre et al., 2016). The presence of several BCA and the observed PPN densities appear indicative of complex rhizosphere interactions, based on the BCA host preference, polyphagy, and other interactions that may affect nematode regulation.

The metabarcoding analysis also showed the occurrence of antagonists of fungi, including *A. luteoalbus* and unclassified species of phyla Rozellomycota and Zygomycota (Supplementary Figures 2A,B). The former has been reported as an antagonist of *Alternaria, Fusarium*, and *Phytophthora* spp. (Lv et al., 2019). It was reported as a causal agent of ginger rhizome rot (Moreira et al., 2013), as an antagonist of fungi and a mushroom pathogen (He et al., 2010; Zhang and Tang, 2015), and as an endophytic plant growth promoter (Khalmuratova et al., 2021). *Rozellomycota* include mostly unclassified zoosporic species characterized by thick-walled resting spores and obligate parasitism on protozoa and fungi (Doweld, 2013). They were found almost uniquely in the banana rhizosphere (Figures 7, 8). Although the biology of these taxa is still poorly investigated, a natural regulatory role may be assigned to members of these clades, which include endoparasitic species in fungi and other eukaryotes. Their density and frequency in banana samples may have been favored by the organic matter applied to plants and by irrigation, providing beneficial services in the rhizosphere such as carbon and nutrients recycling, as well as the regulation of fungal root pathogens.

Finally, the effect of cropping regimes on microbial community structures is considered to be more effective over a longer cultivation period, resulting more evident in older crops or plants (Granzow et al., 2017). Farm age, however, did not show significant differences in the sequence representations (%) of fungi, apart from *Wallemia*, a basidiomycete genus including species inhabiting highly osmotic environments including dry and hypersaline substrates (Zajc and Gunde-Cimerman, 2018), more represented in farms >40 years old (Supplementary Table 6).

## Conclusions

The comparison of microbiota composition between banana rhizosphere samples and close, adjacent controls deprived of banana roots showed differences affecting both bacterial and fungal profiles, indicating an effect of cropping. When considering other variables, sample clusterings also reflected latitude effects for both bacteria and fungi. Metabarcoding data showed the occurrence of taxa reported as BCA of nematodes, as well as other endophytes, mycorrhizal species, and obligate endoparasitic taxa (i.e., Rozellomycota), almost only present in the banana samples. However, apart from *a Dactylella* spp., the nematophagous fungi did not show a strict association or correlation with the two PPN species found, *P. goodeyi* and *Helicotylenchus* spp. Instead, differences were found among the nematode guilds as each phytoparasitic, free-living, and predatory nematode group showed correlations with a specific and different subset of bacteria and fungi. Other factors considered, such as crop cultivation method and soil texture, showed differences in fungal sequence representations as a function of the different variables examined. They were mostly related to trophic specialization and specific biotic requirements or adaptations to a range of decomposers, beneficial endophytes, mycorrhizae, or BCA, as well as plant pathogens. In conclusion, the belowground bacterial and fungal microbiota profiles were affected by plants and latitude, and showed different links to the nematode taxa present. As the impact of microbial species depends not only on their relative but also on their absolute abundance, direct, and specific quantification measurements of the rhizosphere microbial loads for most differential taxa may result informative to exploit the nematode and BCA interactions detected.

## Data Availability Statement

The datasets produced in this study can be found in the following online repository: https://www.ncbi.nlm.nih.gov/, SRA BioProject PRJNA540248.

## Author Contributions

AC, MC, and LR planned and designed the research work. JL-C, MC, and LR performed local samplings. MC performed RNA extraction, bioinformatic work, and the production of sequence data sets. AC and MC interpreted and analyzed the data and wrote this manuscript. All authors critically reviewed, revised, and approved the final version of this manuscript.

## Funding

Research funded by EU H2020 Project MUSA, Microbial uptakes for sustainable management of major banana pests and diseases, GA n. 727624.

## Conflict of Interest

The authors declare that the research was conducted in the absence of any commercial or financial relationships that could be construed as a potential conflict of interest.

## Publisher's Note

All claims expressed in this article are solely those of the authors and do not necessarily represent those of their affiliated organizations, or those of the publisher, the editors and the reviewers. Any product that may be evaluated in this article, or claim that may be made by its manufacturer, is not guaranteed or endorsed by the publisher.

## Footnotes

^1^ www.igatechnology.com

^2^ https://www.illumina.com/content/dam/illumina-marketing/documents/products/appnotes/16S-Metagenomic-Library-Prep-Guide.pdf

^3^ https://github.com/neufeld/pandaseq

^4^ https://biomcs.iimog.org

^5^ http://kiwi.cs.dal.ca/Software/STAMP

^6^*Leff J. W*. (*2016). mctoolsr: Microbial Community Data Analysis Tools. R package version 0.1.0.12*. Available online at: https://github.com/leffj/mctoolsr/.

^7^ http://bioinformatics.psb.ugent.be/webtools/Venn/
