# Supplementary material for: Rhizosphere 16S-ITS Metabarcoding Profiles in Banana Crops Are Affected by Nematodes, Cultivation, and Local Climatic Variations
Source: Front Microbiol. 2022 Jun 9;13:855110. doi: 10.3389/fmicb.2022.855110 (PMC9218937; doi:10.3389/fmicb.2022.855110)
Supplement: Supplementary file 1 [file Table_1.PDF]

Supplementary Table 1. Location and details of sampled farms.

Samples	Latitude/ Longitude	Banana var.	Years of planting	Applied Control	Pests	Products applied
N 1, N2, N3	28°22'00.1"N 16°48'14.6"W	Pequeña Enana	< 5	Biological	*Cosmopolites sordidus*	Pheromone traps
N4, N5, N 6	28°22'01.8"N 16°48'25.2"W	Pequeña Enana	> 50	Conventional	*Cosmopolites sordidus Pratylenchus goodeyi*	Pheromone traps Cloripirifos, Spirodiclofen
N7, N8, N9	28°22'37.0"N 16°44'07.0"W	Pequeña Enana	> 100	Conventional	*Cosmopolites sordidus*	Pheromone traps Cloripirifos, Spirodiclofen
S1, S2, S 3	28°10'07.4"N 16°26'14.6"W	Pequeña Enana	< 5	Integrated	*Nematodes*	Movento, Zeldox, QL-Agri
S4, S5, S6	28°09'20.3"N 16°48'00.6"W	Pequeña Enana	> 40	Integrated	*Cosmopolites sordidus Nematodes*	Indoxicarb, sulfur, pine needles
S7, S8, S9	28°12'25.5"N 16°49'37.2"W	Gruesa	< 5	Biological	-	Pepper extract, potassic soap, sulfur
