# Supplementary material for: Rhizosphere 16S-ITS Metabarcoding Profiles in Banana Crops Are Affected by Nematodes, Cultivation, and Local Climatic Variations
Source: Front Microbiol. 2022 Jun 9;13:855110. doi: 10.3389/fmicb.2022.855110 (PMC9218937; doi:10.3389/fmicb.2022.855110)
Supplement: Supplementary file 2 [file Table_2.PDF]

Supplementary Table 2. Densities of nematodes, pH and soil texture data, per sample.

Sample*	*Pratylenchus goodeyi***	*Helicotylenchus* spp. **	Free living**	Predatory**	Total not parasitic**	pH	Sand (%)	Silt (%)	Clay (%)
N1	-	700	1800	-	1800	8.80	34.0	41.41	24.56
N2	1000	500	1900	-	1900	7.70	33.6	44.69	21.71
N3	900	1200	1100	-	1100	8.40	32.6	40.66	26.73
N4	267	-	533	-	533	7.20	39.6	46.80	13.58
N5	200	400	4100	-	4100	7.00	45.6	39.36	15.02
N6	200	300	1300	200	1500	7.00	33.3	52.30	14.36
N7	-	1600	1800	-	1800	7.80	29.2	46.45	24.35
N8	-	800	900	-	900	7.30	31.4	46.82	21.73
N9	1100	300	3000	-	3000	7.60	38.7	44.80	16.46
S1	-	-	2867	-	2867	7.70	50.2	42.60	7.22
S2	67	400	1067	133	1200	7.40	55.5	39.34	5.20
S3	200	500	2800	-	2800	7.50	43.5	51.95	4.56
S4	-	267	3200	-	3200	7.70	50.9	44.98	4.09
S5	-	2300	2600	-	2600	7.70	61.7	33.46	4.81
S6	-	700	2300	-	2300	7.50	53.4	41.71	4.88
S7	-	-	4900	-	4900	7.10	51.7	43.75	4.55
S8	-	-	2400	100	2500	7.60	48.8	47.66	3.55
S9	-	-	13533	-	13533	8.50	56.1	42.18	1.68
NC1	-	-	2640	300	2940	7.25	54.3	44.31	1.36
NC2	-	-	467	-	467	7.30	79.7	19.27	1.02
NC3	-	-	480	60	540	7.40	83.5	16.17	0.28
NC4	-	-	2820	360	3180	7.40	53.3	45.78	0.90
NC5	-	60	420	-	420	7.25	54.2	45.44	0.37
NC6	-	-	1467	333	1800	7.26	72.5	26.78	0.70
NC7	-	-	2200	-	2200	7.00	66.7	29.74	3.59
NC8	-	-	120	-	120	7.40	77.5	19.07	3.39
NC9	-	-	600	133	733	7.10	74.4	24.85	0.77
SC1	-	-	0	-	-	7.70	82.7	16.86	0.44
SC2	-	-	0	133	133	7.30	83.9	15.66	0.45
SC3	-	-	0	-	-	7.50	76.2	21.84	1.93
SC5	-	-	0	-	-	7.50	64.5	30.84	4.70
SC6	-	-	133	-	133	7.80	88.7	9.35	1.94
SC7	-	-	0	-	-	6.40	59.0	34.76	6.27
SC8	-	-	0	-	-	6.30	66.5	28.97	4.50
SC9	-	-	0	-	-	7.20	80.9	16.37	2.71
M1	-	-	453	-	453	7.70	41.1	50.52	8.36
M2	1750	1000	1050	-	1050	7.00	36.6	51.44	11.94

* Samples code: N = banana, north; NC = control, north; S= banana, south; SC= control, south; M = banana, north.

** Nematodes · 100 cc soil $^{-1}$ (all stages).
