# Supplementary material for: Rhizosphere 16S-ITS Metabarcoding Profiles in Banana Crops Are Affected by Nematodes, Cultivation, and Local Climatic Variations
Source: Front Microbiol. 2022 Jun 9;13:855110. doi: 10.3389/fmicb.2022.855110 (PMC9218937; doi:10.3389/fmicb.2022.855110)
Supplement: Supplementary file 3 [file Table_3.PDF]

Supplementary Table 3. Results of PERMANOVA pairwise comparisons of 16S sequence representation in samples grouped by different classification variables (n = 37, based on Bray-Curtis dissimilarity matrix).

Var 1	Var 2	R^2	P	P Bonferroni	P FDR[a]
Crop and Latitude					
Banana, North	Banana, South	0.08278352	0.018	0.108	**0.018**
Banana, North	Other, North	0.32415512	0.001	0.006	**0.002**
Banana, North	Other, South	0.29550512	0.001	0.006	**0.002**
Banana, South	Other, North	0.42212657	0.001	0.006	**0.002**
Banana, South	Other, South	0.38172876	0.001	0.006	**0.002**
Other, North	Other, South	0.25173629	0.001	0.006	**0.002**
Latitude					
North	South	0.05923199	0.038	0.038	**0.038**
Crop					
Banana	Other	0.2702214	0.001	0.001	**0.001**
Crop, Latitude and type of farm					
Banana, N, Conventional	Banana, N, Organic	0.2159430	0.010	0.280	**0.023**
Banana, N, Conventional	Banana, S, Conventional	0.1341645	0.009	0.252	**0.027**
Banana, N, Conventional	Banana, S, Organic	0.1499247	0.025	0.700	**0.039**
Banana, N, Conventional	Other, N, Conventional	0.3458234	0.002	0.056	**0.037**
Banana, N, Conventional	Other, N, Organic	0.4206565	0.008	0.224	**0.028**
Banana, N, Conventional	Other, S, Conventional	0.3230082	0.002	0.056	**0.037**
Banana, N, Conventional	Other, S, Organic	0.3347764	0.007	0.196	**0.028**
Banana, N, Organic	Banana, S, Conventional	0.2402442	0.023	0.644	**0.040**
Banana, N, Organic	Banana, S, Organic	0.3593641	0.100	2.800	0.114
Banana, N, Organic	Other, N, Conventional	0.4124662	0.004	0.112	**0.025**
Banana, N, Organic	Other, N, Organic	0.6247909	0.100	2.800	0.114
Banana, N, Organic	Other, S, Conventional	0.4278674	0.024	0.672	**0.040**
Banana, N, Organic	Other, S, Organic	0.4982420	0.100	2.800	0.114
Banana, S, Conventional	Banana, S, Organic	0.1869027	0.072	2.016	0.096
Banana, S, Conventional	Other, N, Conventional	0.4395402	0.003	0.084	**0.028**
Banana, S, Conventional	Other, N, Organic	0.5727212	0.012	0.336	**0.024**
Banana, S, Conventional	Other, S, Conventional	0.4378174	0.004	0.112	**0.025**
Banana, S, Conventional	Other, S, Organic	0.4661500	0.009	0.252	**0.027**
Banana, S, Organic	Other, N, Conventional	0.4269341	0.010	0.280	**0.023**
Banana, S, Organic	Other, N, Organic	0.6570915	0.100	2.800	0.114
Banana, S, Organic	Other, S, Conventional	0.4055025	0.036	1.008	0.052
Banana, S, Organic	Other, S, Organic	0.4787059	0.100	2.800	0.114
Other, N, Conventional	Other, N, Organic	0.2337987	0.036	1.008	0.052

Other, N, Conventional	Other, S, Conventional	0.2650999	0.005	0.140	**0.023**
Other, N, Conventional	Other, S, Organic	0.3476636	0.010	0.280	**0.023**
Other, N, Organic	Other, S, Conventional	0.3277487	0.018	0.504	**0.034**
Other, N, Organic	Other, S, Organic	0.5070365	0.100	2.800	0.114
Other, S, Conventional	Other, S, Organic	0.1440523	0.359	10.052	0.359
Crop description					
Other	Gruesa	0.2022546	0.002	0.006	**0.003**
Other	Peq. Enana	0.2728221	0.001	0.003	**0.003**
Gruesa	Peq. Enana	0.0741305	0.054	0.162	0.054
Farm company					
Ar	Arn	0.11664415	0.217	4.557	0.304
Ar	Cplc	0.20221062	0.199	4.179	0.298
Ar	Eds	0.15790715	0.142	2.982	0.398
Ar	Kta	0.11617362	0.183	3.843	0.320
Ar	Mlp	0.13737875	0.165	3.465	0.315
Ar	Mdrs	0.09722896	0.300	6.300	0.332
Arn	Cplc	0.19477020	0.187	3.927	0.302
Arn	Eds	0.14589915	0.109	2.289	0.458
Arn	Kta	0.12078193	0.142	2.982	0.398
Arn	Mlp	0.09561563	0.315	6.615	0.331
Arn	Mdrs	0.12886316	0.121	2.541	0.424
Cplc	Eds	0.10998241	1.000	21.000	1.000
Cplc	Kta	0.23544247	0.145	3.045	0.338
Cplc	Mlp	0.21778338	0.236	4.956	0.310
Cplc	Mdrs	0.15958010	0.253	5.313	0.295
Eds	Kta	0.21982298	0.038	0.798	0.798
Eds	Mlp	0.17847810	0.065	1.365	0.341
Eds	Mdrs	0.11421904	0.245	5.145	0.303
Kta	Mlp	0.13378128	0.149	3.129	0.313
Kta	Mdrs	0.17371477	0.061	1.281	0.640
Mlp	Mdrs	0.16415595	0.064	1.344	0.448
pH [a]					
MAL	N	0.05977778	0.7960000	7.960000	0.995
MAL	SAC	0.67819662	0.3333333	3.333333	0.741
MAL	SAL	0.36359520	1.0000000	10.000000	1.000
MAL	SLAL	0.03563508	0.8880000	8.880000	0.987
N	SAC	0.10332296	0.1040000	1.040000	0.520
N	SAL	0.08961877	0.2450000	2.450000	0.817
N	SLAL	0.03047690	0.4290000	4.290000	0.715

SAC	SAL	0.51611773	0.3333333	3.333333	0.741
SAC	SLAL	0.08589264	0.0710000	0.710000	0.710
SAL	SLAL	0.04900152	0.4790000	4.790000	0.684
Pratylenchus [b]					
L	M	0.04722871	0.119	0.357	0.357
L	VH	0.04600486	0.135	0.405	0.202
M	VH	0.16002623	0.240	0.720	0.240
Helicotylenchus [c]					
H	L	0.06655667	0.0490000	0.294	0.147
H	M	0.07207040	0.8770000	5.262	0.877
H	VH	0.36673465	0.3333333	2.000	0.500
L	M	0.10689535	0.0010000	0.006	**0.006**
L	VH	0.05371559	0.1860000	1.116	0.372
M	VH	0.08097848	0.7080000	4.248	0.850
Type of farm					
Conventional	Integrated	0.05803276	0.129	0.387	0.387
Conventional	Organic	0.05646789	0.142	0.426	0.213
Integrated	Organic	0.04618275	0.353	1.059	0.353
Free living nematodes [d]					
H	L	0.05568622	0.2870000	1.722	0.861
H	M	0.07201434	0.3920000	2.352	0.784
H	VH	0.49171363	0.6666667	4.000	0.800
L	M	0.07161939	0.0210000	0.126	0.126
L	VH	0.04783304	0.4990000	2.994	0.748
M	VH	0.06770509	0.7410000	4.446	0.741
Predatory nematodes [e]					
L	M	0.04722871	0.119	0.357	0.357
L	VH	0.04600486	0.122	0.366	0.183
M	VH	0.16002623	0.218	0.654	0.218
Age					
<5	>40	0.05231987	0.061	0.061	0.061

* Significant values are shown in bold on yellow background.
[a] pH range: MAL = moderately alkaline (7.9–8.4); N = neutral (6.6–7.3); SAC = slightly acid (6.1–6.5); SAL = strongly alkaline (8.5–9.0); SLAL = slightly alkaline (7.4–7.8).
[b] Nematodes / 100 cc soil. L = low or absent (0-150); M = medium (151-533); H = high (534-916); VH = very high (> 516). Overall mean ± SD = 150 ± 383.
[c] Nematodes / 100 cc soil. L = low or absent (0-290); M = medium (291-804); H = high (805-1319); VH = very high (> 1319). Overall mean ± SD = 290 ± 514.
[d] Nematodes / 100 cc soil. L = low or absent (0-50); M = medium (51-148); H = high (149-247); VH = very high (> 247). Overall mean ± SD = 50 ± 99.
[e] Nematodes / 100 cc soil. L = low or absent (0-50); M = medium (51-148); H = high (149-247); VH = very high (> 247). Overall mean ± SD = 50 ± 99.
