# Supplementary material for: Rhizosphere 16S-ITS Metabarcoding Profiles in Banana Crops Are Affected by Nematodes, Cultivation, and Local Climatic Variations
Source: Front Microbiol. 2022 Jun 9;13:855110. doi: 10.3389/fmicb.2022.855110 (PMC9218937; doi:10.3389/fmicb.2022.855110)
Supplement: Supplementary file 4 [file Table_4.PDF]

Supplementary Table 4. Frequencies of fungi belonging to genera with known nematophagous or other biocontrol activity between banana rhizosphere soil and adjacent controls, as shown by ITS2 sequence number repartitions.

Fungal taxa	Banana rhizosphere		Other samples (controls)		Number of sequences [c]	
	Sequence numbers [a]	Samples (%) [b]	Sequence numbers [a]	Samples (%) [b]	Total	range
Acrostalagmus luteoalbus	359.4 ± 246.4	20 (100)	187.8 ± 89.4	12 (70.5)	9441	1 - 5001
Arthrobotrys amerospora	60.7 ± 28.7	3 (15.0)	-	-	182	14 - 113
Arthrobotrys conoides	-	-	7.0	1 (5.8)	7	7
Arthrobotrys oligospora	174.5 ± 79.5	8 (40.0)	5.0 ± 1.0	3 (17.6)	1411	3 - 636
Arthrobotrys scaphoides	-	-	3.0	2 (11.7)	6	3
Arthrobotrys sp.	204.1 ± 67.5	9 (45.0)	30.8 ± 21.7	6 (35.2)	2022	1- 698
Arthrobotrys superba	34.6 ± 15.5	7 (35.0)	6.5 ± 3.5	2 (11.7)	255	1 - 110
Beauveria sp.	31.0 ± 25.1	6 (30.0)	6.0	1 (5.8)	192	1 - 156
Brachyphoris oviparasitica	-	-	7.4 ± 4.3	5 (29.4)	37	1 - 24
Dactylaria ampulliformis	-	-	4.0	1 (5.8)	4	4
Dactylella atractoides	108.5 ± 92.5	2 (10.0)	24.5 ± 13.2	6 (35.2)	364	1 - 201
Dactylella heptameres	18.0	1 (5.0)	-	-	18	18
Dactylella intermedia	24.0 ± 12.0	2 (10.0)	-	-	48	12 - 36
Dactylella sp.	136.1 ± 82.0	7 (35.0)	22.0 ± 18.0	2 (11.7)	997	1 - 563
Drechslerella dactyloides	-	-	5.0 ± 4.0	2 (11.7)	10	1 - 9
Duddingtonia flagrans	35.7 ± 14.2	3 (15.0)	-	-	107	10 - 59
Metarhizium anisopliae	31.2 ± 15.4	5 (25.0)	253.8 ± 236.0	4 (23.5)	1171	1 - 961
Nematoctonus concurrens	-	-	15.0	1 (5.8)	15	15
Nematoctonus pachysporus	244.0	1 (5.0)	-	-	244	244
Orbiliaceae uncl.	12.3 ± 5.4	4 (20.0)	17.5 ± 3.0	4 (23.5)	119	4 - 28
Pleurotus ostreatus	27	1 (5.0)	-	-	27	27
Pochonia (Metacordyceps) chlamydosporia	7.7 ± 5.3	3 (15.0)	-	-	23	1 - 14
Trichoderma harzianum/ Hypocrea lixii	10.9 ± 10.0	11 (55.0)	8.0	1 (5.8)	286	1 - 110
Trichoderma longibrachiatum	3 ± 1.0	2 (10.0)	4.3 ± 1.8	3 (17.6)	19	1 - 7
Trichoderma sp.	40.8 ± 18.5	12 (60.0)	1	4 (23.5)	494	1 - 212
Trichoderma virens	-	-	1	1 (5.8)	1	1

[a] Mean from positive samples ± SE. [b] Number of positive samples (%), per group. [c] Sum and range from all positive samples.
