# Supplementary material for: Rhizosphere 16S-ITS Metabarcoding Profiles in Banana Crops Are Affected by Nematodes, Cultivation, and Local Climatic Variations
Source: Front Microbiol. 2022 Jun 9;13:855110. doi: 10.3389/fmicb.2022.855110 (PMC9218937; doi:10.3389/fmicb.2022.855110)
Supplement: Supplementary file 5 [file Table_5.PDF]

Supplementary Table 5. Distribution of the 429 fungal taxa within the sample groups classified by crop and latitude, as shown by Venn analysis.

**Groups***	**total**	**elements**
Banana North<br>Banana South<br>Control North<br>Control South	94	*Acremonium chrysogenum* FU_U57672, Oedocephalum sp. OTU43966, Hypocreales sp. OTU118036, *Peyronellaea* sp. OTU962325, *Metarhizium anisopliae* OTU1049761, *Fusarium* sp. OTU15, Basidiomycota uncl. OTU414131, Chaetomiaceae uncl. OTU608, Helotiales uncl. OTU1310, *Preussia* sp. OTU1651, *Stagonospora* sp. OTU1124, *Acrostalagmus luteoalbus* OTU470, Orbiliaceae uncl. OTU305098, *Myrothecium* sp. OTU823, *Trichoderma* sp. OTU47978, Coniochaetales sp. FU_JF519325, *Chaetomium* sp. OTU131, Ascomycota uncl. OTU1426, *Cyphellophora* sp. OTU912, *Cryptococcus* sp. OTU1001341, Agaricaceae uncl. OTU267159, Dothideomycetes uncl. OTU824, *Mortierella* sp. OTU136, *Acremonium* sp. OTU24332, *Stachybotrys* sp. OTU297886, *Oidiodendron* sp. OTU551360, *Malassezia* sp. OTU100, *Aspergillus calidoustus* OTU1770, Ascobolaceae uncl. OTU3608, Glomeraceae uncl. FU_GU322945, *Arthrobotrys oligospora* OTU553, Trichosphaeriales uncl. OTU269, *Aspergillus penicillioides* OTU769, Pezizaceae uncl. OTU1339, *Macrophomina phaseolina* FU_KF766195, Onygenales uncl. OTU1173, Capnodiales uncl. OTU22763, *Rhizopus arrhizus* OTU1448, Nectriaceae uncl. OTU565, Microascaceae OTU75, *Cladosporium* sp. OTU1425, *Aspergillus* sp. OTU886, Pleosporales uncl. OTU843, *Sporobolomyces* sp. OTU985, Tremellomycetes sp. OTU1673, Spizellomycetaceae sp. OTU1293, Sordariales uncl. OTU1358, Gymnoascaceae sp. OTU1019033, Bionectriaceae uncl. FU_GU973834, Malasseziales uncl. OTU297, *Westerdykella* sp. OTU1663, *Dactylella atractoides* OTU499347, Xylariales uncl. OTU217513, Chaetothyriales uncl. OTU1313, Myxotrichaceae uncl. OTU426, Scytalidium sphaerosporum OTU868023, *Mortierella oligospora* OTU684, *Aspergillus insuetus* OTU287097, *Debaryomyces* sp. FU_AB054023, *Coprinellus* sp. OTU765219, *Conocybe* sp. OTU177291, *Wallemia* OTU1317, *Zopfiella* sp. OTU162745, *Sarocladium* sp. OTU218273, *Alternaria* sp. OTU2021, Rozellomycota uncl. OTU451, *Spizellomyces acuminatus* OTU1067786, Polyporales uncl. OTU62137, *Ceratobasidium* sp. OTU480110, *Clavispora lusitaniae* FU_DQ249208, *Articulospora* sp. OTU1440, *Malassezia globosa* OTU256, Trichocomaceae sp. OTU205, Dothideales uncl. OTU650992, *Stemphylium herbarum* OTU373, Sordariomycetes uncl. OTU850, Pleosporaceae uncl. OTU1623, *Arthrographis* sp. OTU534, *Madurella pseudomycetomatis* OTU26926, Ceratobasidiaceae uncl. OTU41023, *Exophiala lecanii-corni* OTU198084, *Phoma* sp. OTU65469, *Penicillium* sp. OTU851256, *Mortierella alpina* OTU408, Pyronemataceae sp. OTU7711, Tremellales uncl. FU_AF444399, *Podospora* sp. OTU79519, Eurotiales uncl. OTU103325, *Torula caligans* OTU1039, *Chalastospora ellipsoidea* OTU46352, *Arthrobotrys* sp. OTU285, Auriculariales sp. OTU1583, *Stagonosporopsis* sp. OTU748, Pezizomycetes uncl. OTU1319
Banana North<br>Banana South<br>Control North	40	Herpotrichiellaceae uncl. OTU327337, *Acremonium alcalophilum* OTU1001210, *Acremonium alternatum* OTU31567, *Hormonema viticola* OTU436924, *Mucor* sp. OTU769202, *Phialophora geniculata* OTU958462, *Dactylella* sp. OTU1430, *Trichoderma harzianum* OTU978908, *Thermomyces lanuginosus* OTU1375, *Cladophialophora* sp. OTU1406, *Leptodiscella chlamydospora* OTU128075, Chytridiomycota sp. OTU17243, *Plectosphaerella alismatis* OTU90630, Sebacinales sp. OTU311210, *Arthropsis hispanica* OTU717,

		Psathyrella sp. OTU922292, Agaricales uncl. OTU15385, Agaricus sp. OTU221624, *Exophiala* sp. OTU1190, Chytridiomycetes sp. OTU586, *Cryptococcus randhawii* OTU492, *Scytalidium* sp. OTU691314, *Olpidiales* uncl. OTU1346, *Beauveria* sp. OTU122220, *Mortierella ambigua* OTU177, *Talaromyces* sp. OTU217443, *Stachybotrys chartarum* OTU550993, *Petriella* sp. OTU621495, Leotiomycetes uncl. OTU524434, *Musicillium theobromae* OTU665909, *Olpidiaster brassicae* OTU1249, *Ochroconis tshawytschae* FU_FR832476, *Exophiala mesophila* OTU302, *Ilyonectria* sp. OTU390502, *Mortierella amoeboidea* OTU41224, Lasiosphaeriaceae uncl. OTU691, *Leptodiscella* sp. OTU346206, Eurotiomycetes uncl. OTU700822, *Chrysosporium lobatum* OTU443, *Arthrobotrys superba* OTU103231
Banana North<br>Control North<br>Control South	12	*Candida blankii* OTU779, Onygenaceae sp. OTU123612, *Aspergillus sclerotiorum* OTU45362, *Aspergillus niger* OTU31243, *Cryptococcus magnus* OTU1090, *Stemphylium* sp. OTU238778, *Aureobasidium* sp. OTU1627, *Alternaria alternata* OTU300, Dothioraceae uncl. OTU195946, *Microdochium* sp. OTU476651, *Candida* sp. OTU342689, Pezizales uncl. OTU353638
Banana North<br>Banana South<br>Control South	8	*Fomitopsis pinicola* OTU171, *Mycothermus thermophilus* FU_JF412007, *Microascus* sp. OTU242376, *Cunninghamella blakesleeana* OTU257318, *Mortierella capitata* OTU1666, *Emmonsia* sp. OTU1007, *Leucocoprinus cretaceus* OTU576455, *Pseudogymnoascus* sp. OTU915
Banana South<br>Control North<br>Control South	13	Filobasidiales sp. OTU647123, *Cephaliophora tropica* OTU634903, *Sordaria* sp. OTU465841, *Cladorrhinum bulbillosum* OTU595187, *Fusarium brachygibbosum* OTU471238, *Scolecobasidium constrictum* OTU700530, *Phialophora verrucosa* OTU411710, *Aspergillus fumigatus* OTU13833, *Trichosporon* sp. FU_AF444439, Mortierellales sp. OTU246, *Absidia repens* OTU1255, *Humicola* sp. OTU879194, *Trichoderma longibrachiatum* OTU337888
Banana North<br>Control North	28	*Erythrobasidium hasegawianum* OTU246886, *Coniosporium* sp. OTU738332, Catenariaceae uncl. OTU414191, *Candida intermedia* OTU1016573, *Capnodium* sp. OTU709248, *Aspergillus wentii* OTU862137, *Hannaella* sp. OTU783107, *Powellomyces* sp. OTU896595, *Pyrenochaeta lycopersici* OTU20980, *Operculomyces laminatus* OTU620926, *Hyphodontia* sp. OTU214501, *Funneliformis mosseae* FU_HF970250, *Knufia* sp. FU_EU730589, *Paraconiothyrium hawaiiense* OTU653979, *Clathrus ruber* OTU577867, Capnodiaceae uncl. FU_KF826942, *Capronia* sp. OTU53710, Pucciniomycetes uncl. OTU179908, *Minimedusa polyspora* OTU808933, *Acremonium persicinum* OTU675087, *Rhizophlyctis* sp. OTU990627, *Spizellomyces dolichospermus* OTU436051, *Funneliformis* sp. FU_HF970285, *Cyphellophora fusarioides* OTU64788, *Cyphellophora vermispora* OTU775531, *Zygomycota* sp. OTU280230, *Suillus* sp. OTU50400, *Thanatephorus cucumeris* OTU626

Banana North<br>Banana South	23	*Arachnomyces gracilis* OTU936, *Duddingtonia flagrans* OTU411952, *Acremonium charticola* OTU9645, *Gliomastix luzulae* OTU856523, *Chloridium* sp. OTU40398, *Phialocephala xalapensis* OTU603202, *Cephalotrichiella penicillata* OTU914206, *Thielavia intermedia* OTU170933, *Septofusidium herbarum* OTU267335, *Claviceps* sp. OTU484742, *Microsporum* sp. OTU19725, *Arthrographis kalrae* OTU1736, *Exophiala moniliae* OTU400481, *Arthrobotrys amerospora* OTU962550, *Sagenomella* sp. OTU74800, *Metacordyceps chlamydosporia* OTU779793, Clavicipitaceae uncl. OTU24, *Hypocrea lixii* OTU78855, *Chrysosporium* sp. OTU130592, Saccharomycetales FU_AY349453, *Melanophyllum haematospermum* OTU147283, *Umbelopsis* sp. OTU627674, *Phialophora japonica* OTU877029
Banana North<br>Control South	7	*Sterigmatomyces halophilus* OTU680778, *Scedosporium prolificans* OTU1198, *Monodictys* sp. OTU86064, *Pseudallescheria* sp. FU_AJ888414, *Hortaea thailandica* OTU18550, Tulasnellaceae uncl. OTU205725, Cordycipitaceae uncl. OTU688716
Banana South<br>Control North	15	*Setophaeosphaeria* sp. OTU931918, *Lichtheimia ramosa* OTU675024, *Cladophialophora chaetospira* OTU87019, *Stachybotrys echinata* OTU963863, *Scutellinia* sp. OTU678218, *Trichothecium* sp. OTU677767, *Peniophora* sp. OTU1783, *Penicillium georgiense* OTU282790, *Retroconis* sp. OTU112855, Mycosphaerellaceae FU_JX143632, Hypocreaceae sp. OTU277237, *Veronaea* sp. OTU1204, *Paecilomyces* sp. OTU1029245, Verrucariales uncl. OTU75067, *Exobasidium* sp. FU_DQ682574
Control North<br>Control South	28	*Aspergillus terreus* OTU911371, *Brachyphoris oviparasitica* FU_DQ494373, *Curvularia* sp. FU_HE861850, *Lecanicillium* sp. OTU296511, *Polyplosphaeria* sp. OTU953838, *Laetisaria arvalis* OTU987633, *Actinomucor elegans* FU_JN205828, *Aureobasidium pullulans* FU_AF121282, *Pyrenochaeta* sp. OTU1048029, *Aspergillus flavus* FU_KF932304, *Diversispora* sp. OTU4990, *Cryptococcus albidus* OTU191488, Amphisphaeriaceae sp. OTU476127, Leptosphaeriaceae uncl. OTU510356, *Cunninghamella echinulata* FU_GQ221208, *Arthrobotrys scaphoides* OTU488417, *Rhodotorula* sp. FU_JF706656, Mycosphaerellaceae sp. OTU15186, *Wallemia ichthyophaga* OTU888250, *Myrothecium gramineum* OTU773278, Microascales uncl. FU_EU520619, *Fusarium delphinoides* OTU480684, *Phaeotheca triangularis* OTU90, *Cryptococcus laurentii* FU_JQ247574, *Geminibasidium* sp. OTU75977, *Peziza* sp. OTU928327, *Oedocephalum adhaerens* OTU528, Psathyrellaceae uncl. OTU730
Banana South<br>Control South	11	*Spiromastix* sp. OTU1120, Cystofilobasidiaceae uncl. OTU641, *Remersonia thermophila* OTU1034640, Trechisporales sp. OTU561, Agaricomycetes uncl. OTU375007, *Filobasidium floriforme* OTU91286, Mytilinidiaceae uncl. OTU71993, *Scolecobasidium* sp. OTU434856, *Erysiphe* sp. OTU752046, *Wallemia hederae* OTU45181, *Chaetomium cupreum* OTU448620
Banana North	33	*Yunnania penicillata* OTU414676, *Dactylella intermedia* OTU1055171, *Tetracladium* sp. OTU727063, *Ceratocystis* sp. FU_KF863801, *Phialosimplex chlamydosporus* OTU285036, Magnaporthaceae uncl. OTU296188, Teloschistaceae uncl. OTU862020, *Torulaspora delbrueckii* FU_EF568083, *Torulaspora pretoriensis* OTU281913, *Microascus brevicaulis* FU_AJ853776, *Mortierella exigua* OTU443685,

		Claroideoglomus sp. OTU752211, *Cystolepiota cystophora* OTU933482, Lecanoromycetes uncl. OTU446, *Haptocillium campanulatum* OTU596919, *Phialosimplex caninus* OTU299325, *Aplosporella* sp. FU_EF591926, *Blumeria graminis* FU_AB273563, *Podospora pyriformis* OTU170219, *Malbranchea* sp. FU_HE974452, Chytridiaceae uncl. FU_EU754999, Hydnodontaceae sp. OTU727123, Dipodascaceae uncl. FU_KF493938, *Cladosporium halotolerans* OTU151704, *Pleurotus ostreatus* OTU280, *Gibberella fujikuroi* OTU596783, *Trechispora* sp. OTU264588, *Acremonium longisporum* OTU1010303, *Meyerozyma guilliermondii* FU_EF568003, *Galerina* sp. OTU101726, *Lycoperdon* sp. OTU756809, *Acaulospora spinosissima* OTU465536, Stephanosporaceae uncl. OTU282150
Control North	69	*Verticillium* sp. FU_JN188016, *Arthrobotrys conoides* OTU847660, *Exophiala xenobiotica* OTU445912, *Stachybotrys microspora* FU_AF081476, *Rhizomucor miehei* FU_HM999959, *Clitopilus* sp. OTU26562, *Cladosporium flabelliforme* OTU229211, *Cryptococcus dimennae* OTU551061, *Rhizophlyctis rosea* OTU47384, *Auxarthron concentricum* OTU720205, Claroideoglomeraceae OTU198928, *Verrucaria muralis* FU_EU249487, *Myriococcum thermophilum* OTU890375, *Schizothecium* sp. OTU69609, *Ganoderma* sp. OTU556945, *Conocybe apala* OTU529069, *Nematoctonus concurrens* OTU151351, *Cladorrhinum* sp. OTU1032819, *Colletotrichum* sp. OTU879340, *Basidiobolus ranarum* OTU832218, Peniophoraceae uncl. OTU1017123, *Cladosporium ramotenellum* OTU851839, *Papulaspora equi* OTU625688, *Tremella* sp. FU_FN428949, *Coniochaeta* sp. OTU452995, *Circinella* sp. OTU66856, *Rhinocladiella* sp. OTU899562, *Dioszegia* sp. FU_AY562160, *Sarocladium implicatum* OTU300602, Coniella fragariae FU_AY339317, *Lepiota lilacea* OTU52333, *Ganoderma resinaceum* OTU829954, *Caloplaca* sp. OTU205655, *Diaporthe* sp. FU_GU934561, Sporormiaceae sp. OTU275582, *Periconia* sp. OTU127369, *Kochiomyces* sp. OTU133797, *Mariannaea* sp. OTU830846, *Pleonectria zanthoxyli* OTU825009, *Glomus* sp. OTU864960, *Myrothecium roridum* OTU232009, *Pseudaleuria* sp. OTU742871, *Amauroascus kuehnii* OTU141144, *Entoloma* sp. OTU427709, *Yarrowia lipolytica* FU_DQ668345, *Coniothyrium* sp. OTU410807, Glomerales uncl. OTU620499, *Paraconiothyrium* sp. OTU761044, *Lichtheimia corymbifera* OTU90218, *Syncephalastrum racemosum* OTU453943, Trechisporales uncl. OTU138860, Phaeosphaeriaceae uncl. OTU371879, *Leucoagaricus meleagris* OTU575077, *Stachybotrys nephrospora* OTU641220, Lichtheimiaceae uncl. OTU686275, *Sporormia fimetaria* OTU149935, *Rhizophagus* sp. OTU792808, *Dendryphion* sp. FU_KJ869145, Verrucariaceae uncl. OTU253389, *Cryptococcus chernovii* OTU183708, Geastraceae sp. OTU36662, *Catenaria anguillulae* OTU352768, *Rhizophagus irregularis* OTU121080, *Neofusicoccum* sp. FU_AY339262, *Lophiostoma* sp. OTU429732, *Arthrobotrys dactyloides* FU_KJ938574, *Lophiostoma macrostomum* FU_GU827614, *Gymnoascus* sp. OTU113875, *Geastrum floriforme* OTU561634

Banana South	28	*Leucocoprinus cepistipes* FU_UDB016245, *Lepiota revelata* OTU709236, *Ochroconis* sp. OTU217707, *Penicillium spinulosum* OTU613205, *Conocybe deliquescens* FU_FJ362027, *Nematoctonus pachysporus* OTU341407, *Thielavia basicola* OTU24297, *Acremonium restrictum* OTU298346, *Penicillium dierckxii* OTU40671, *Cladorrhinum phialophoroides* OTU161844, *Kockovaella* sp. OTU466043, *Spencermartinsia* sp. FU_KC898225, Massarinaceae sp. OTU1041653, *Blastobotrys proliferans* FU_FM178324, *Candida palmioleophila* OTU163831, *Xylodon sambuci* OTU916696, *Penicillium parviverrucosum* OTU17260, *Fellomyces* sp. FU_AF444337, *Dactylella heptameres* OTU212517, *Thielavia* sp. OTU723891, *Venturia* sp. OTU36096, *Infundichalara* sp. FU_HE603986, *Spiromastix princeps* OTU805837, *Powellomyces hirtus* OTU339105, *Penicillium chermesinum* OTU1004458, *Geomyces auratus* OTU162356, *Dictyosporium* sp. OTU424663, *Penicillium toxicarium* OTU475418
Control South	20	*Xenobotrytis acaducospora* OTU581304, *Udeniomyces pannonicus* OTU625154, Bolbitiaceae uncl. OTU52410, *Aspergillus unguis* OTU716956, *Acremonium nepalense* OTU722324, *Sporidiobolales* uncl. OTU183892, *Corollospora* sp. OTU917773, *Phoma multirostrata* OTU522027, *Neophaeosphaeria* sp. OTU132437, *Coniothyrium multiporum* FU_JF740187, *Leptosphaeria* sp. OTU357261, *Simplicillium* sp. OTU335615, *Hohenbuehelia portegna* OTU432292, *Dactylaria ampulliformis* FU_AY265336, *Aspergillus japonicus* OTU631424, Bolbitiaceae sp. OTU126131, *Trichoderma virens* OTU786607, *Allomyces anomalus* OTU8964, *Cylindrocladiella* sp. FU_JN100643, *Sarcinomyces* sp. OTU904370

* Number of unique elements per group = Banana, north: 245; Banana, south: 232; Control, north: 299; Control, south: 193. "FU_" shows closest NCBI sequence.
