# Supplementary material for: Rhizosphere 16S-ITS Metabarcoding Profiles in Banana Crops Are Affected by Nematodes, Cultivation, and Local Climatic Variations
Source: Front Microbiol. 2022 Jun 9;13:855110. doi: 10.3389/fmicb.2022.855110 (PMC9218937; doi:10.3389/fmicb.2022.855110)
Supplement: Supplementary file 6 [file Table_6.PDF]

Supplementary Table 6. Taxa summary for more represented fungi at the family or genus levels, analyzed comparing samples grouped by crop and latitude and other classification variables, filtered for sequence representations (%) ≥ 5% in at least one group (unless otherwise specified). Significant comparisons among sample groups are shown in bold (Kruskal-Wallis t-test, P ≤ 0.05) with highest sequence representations (%) in red. Data analyzed with R library *mctoolsr*.

Taxa (family)	P	P Bon	P FDR	Crops and latitude			
				Banana_North	Banana_South	Other_North	Other_South
Wallemiaceae	**0.0002**	**0.0020**	**0.0020**	0.1235	0.0655	0.0012	0.0001
Glomeraceae	**0.0003**	**0.0034**	**0.0017**	0.0036	0.0005	0.0776	0.0053
Microascaceae	**0.0013**	**0.0154**	**0.0051**	0.0789	0.0870	0.0285	0.0025
Clavicipitaceae	**0.0076**	0.0912	**0.0228**	0.0083	0.0040	0.0265	0.0712
Agaricaceae	**0.0077**	0.0928	**0.0186**	0.1883	0.1170	0.0370	0.3020
Claroideoglomeraceae	**0.0095**	0.1136	**0.0189**	0.0686	0.0066	0.0816	0.0798
Lasiosphaeriaceae	**0.0131**	0.1572	**0.0225**	0.0312	0.0284	0.0703	0.0061
Bolbitiaceae	**0.0174**	0.2087	**0.0261**	0.0286	0.0503	0.1092	0.0390

Taxa (genus)	P	P Bon	P FDR	Crops and latitude			
				Banana_North	Banana_South	Other_North	Other_South
Wallemia	0.000166	0.001662	**0.001662**	0.123530	0.065454	0.001235	0.00007
Glomeraceae uncl.	0.000182	0.001824	**0.000912**	0.002858	0.000436	0.076656	0.00503
Hypocreaceae uncl.	0.000820	0.008196	**0.002732**	0.061698	0.02273	0.019548	0.00002
Cystolepiota uncl.	0.001862	0.018617	**0.004654**	0.166215	0.109007	0.014213	0.28800
Metarhizium	0.002936	0.02936	**0.005872**	0.008019	0.001733	0.025795	0.06810
Petriella	0.003193	0.031935	**0.005322**	0.077459	0.073828	0.027755	0.00197
Claroideoglomeraceae uncl.	0.006959	0.069585	**0.009941**	0.068211	0.005688	0.051524	0.04550
Bolbitiaceae uncl.	0.039778	0.397784	**0.049723**	0.027282	0.038974	0.104051	0.03550

Taxon (genus)	P	P Bon	P FDR	Farm age (years)	
				<5	>40
Wallemia	**0.009556**	0.057335	**0.057335**	0.006842	0.096655

Taxa	P	P Bon	P FDR	Crop type		
				Conventional	Integrated	Organic
Hypocreaceae uncl.	**0.029804**	**0.238435**	**0.238435**	0.054985	0.018462	0.007207

Taxon (genus)	P	P Bon	P FDR	Crop type (seq. threshold > 1 %)		
				Conventional	Integrated	Organic
Arthrobotrys	**0.00126**	0.05054	**0.05054**	0.01100	0.03484	0.00111

Taxon (genus)	P	P Bon	P FDR	*Pratylenchus* spp. (seq. threshold >1 %) [a]		
				L	M	VH
Diaporthe	0.000094	0.003288	**0.003288**	0.000002	0.018016	0.000000

[a] Based on nematodes / 100 cc soil. L = low or absent (0-150); M = medium (151-533); H = high (534-916); VH = very high (> 516). Overall mean ± SD = 150 ± 383.

Taxa	P	P Bon	P FDR	*Helicotylenchus* spp. (seq. threshold >1 %) [b]			
				L	M	H	VH
Wallemia	0.000677	0.02574	**0.02574**	0.009240	0.100110	0.089203	0.3292119
Coniochaeta	0.001139	0.043287	**0.021644**	0.004682	0.015195	0.009187	0.011688
Powellomyces	0.003662	0.139155	**0.046385**	0.000697	0.020983	0.007231	0.0034854

[b] Based on nematodes / 100 cc soil. L = low or absent (0-290); M = medium (291-804); H = high (805-1319); VH = very high (> 1319). Overall mean ± SD = 290 ± 514.

Taxa	P	P Bon	P FDR	pH (seq. threshold >1 %) [c]				
				MAL	N	SAC	SAL	SLAL
Diaporthe	0.000020	0.000855	**0.000855**	0.018016	0.000000	0.000000	0.000035	0.000000
Zygomycota uncl.	0.000998	0.042905	**0.021453**	0.000000	0.000000	0.029762	0.000052	0.000002

[c] pH range: MAL = moderately alkaline (7.9–8.4); N = neutral (6.6–7.3); SAC = slightly acid (6.1–6.5); SAL = strongly alkaline (8.5–9.0); SLAL = slightly alkaline (7.4–7.8).
