# Supplementary material for: Rhizosphere 16S-ITS Metabarcoding Profiles in Banana Crops Are Affected by Nematodes, Cultivation, and Local Climatic Variations
Source: Front Microbiol. 2022 Jun 9;13:855110. doi: 10.3389/fmicb.2022.855110 (PMC9218937; doi:10.3389/fmicb.2022.855110)

Supplementary Figure 1. Relationship between densities of *Helicotylenchus* spp. and *Pratylenchus goodeyi*, in sampled soils.

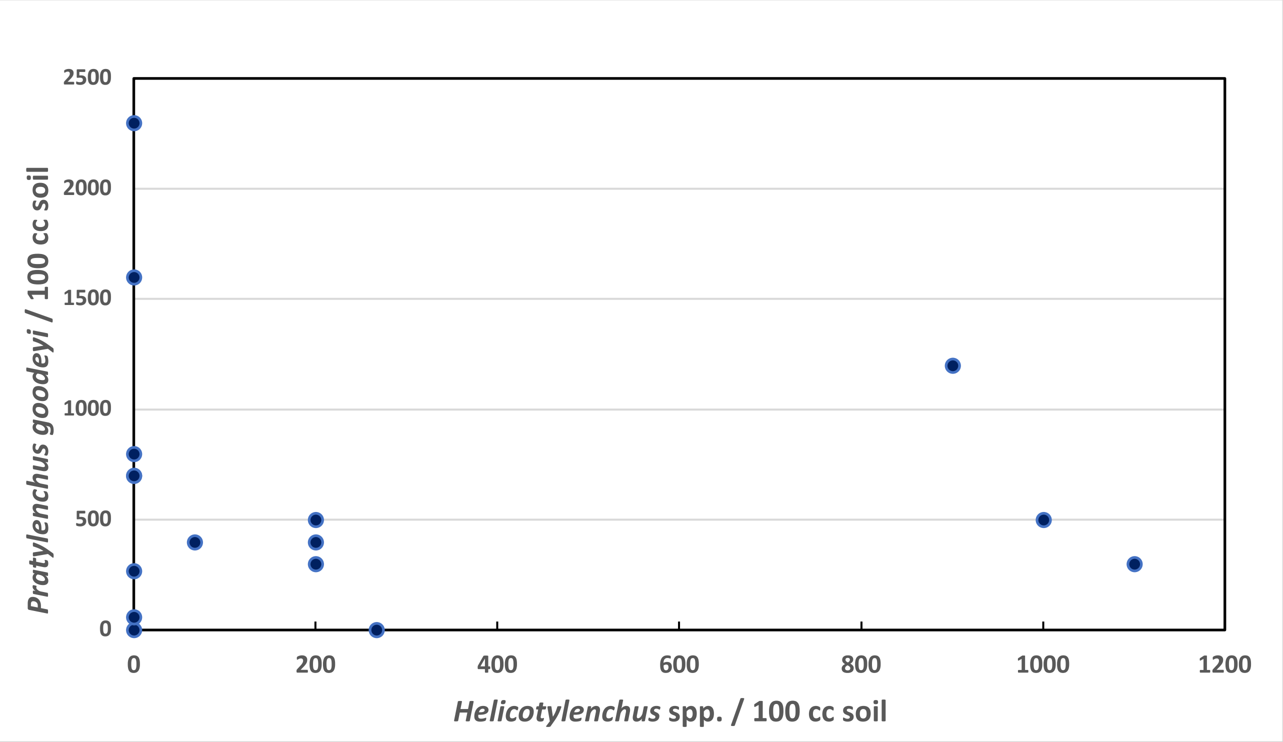

Supplement: Supplementary file 9 [file Image_1.pdf]
