# Supplementary material for: Rhizosphere 16S-ITS Metabarcoding Profiles in Banana Crops Are Affected by Nematodes, Cultivation, and Local Climatic Variations
Source: Front Microbiol. 2022 Jun 9;13:855110. doi: 10.3389/fmicb.2022.855110 (PMC9218937; doi:10.3389/fmicb.2022.855110)

Supplementary Figure 2. Abundance of ITS sequences in samples from banana and controls, at the phylum level (A) and, at the class level, in samples classified by crop and latitude (B). Hierarchical cluster analysis of samples by ITS sequence abundance, classified by crop type and presence of banana plants (left) or latitude (C).

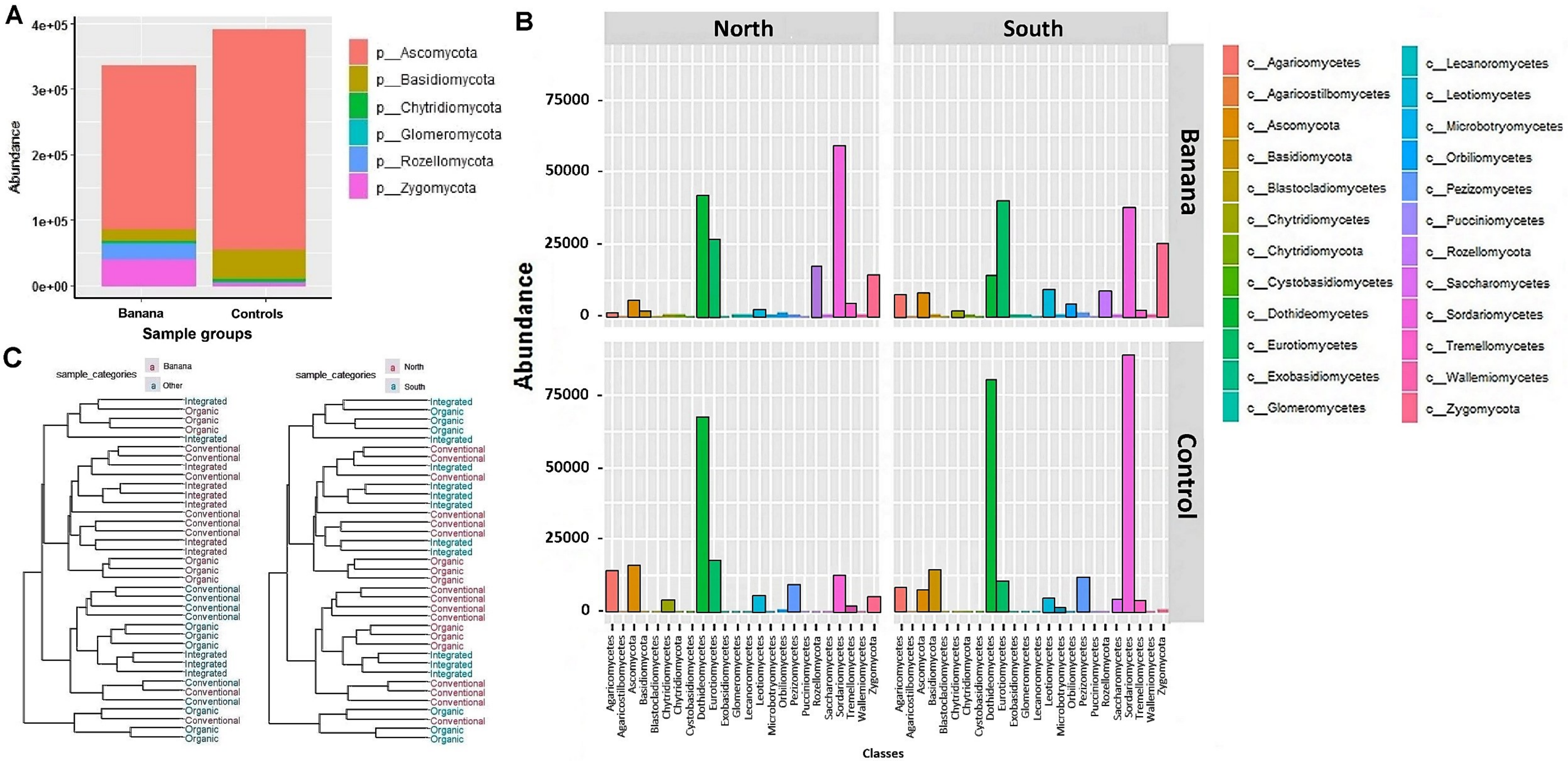

Supplement: Supplementary file 10 [file Image_2.pdf]
