# Supplementary material for: Rhizosphere 16S-ITS Metabarcoding Profiles in Banana Crops Are Affected by Nematodes, Cultivation, and Local Climatic Variations
Source: Front Microbiol. 2022 Jun 9;13:855110. doi: 10.3389/fmicb.2022.855110 (PMC9218937; doi:10.3389/fmicb.2022.855110)

Supplementary Figure 3. Bar plots based on ITS sequence data showing differential abundance of taxa at the order level, in samples grouped by crop and latitude (horizontal bars show group means).

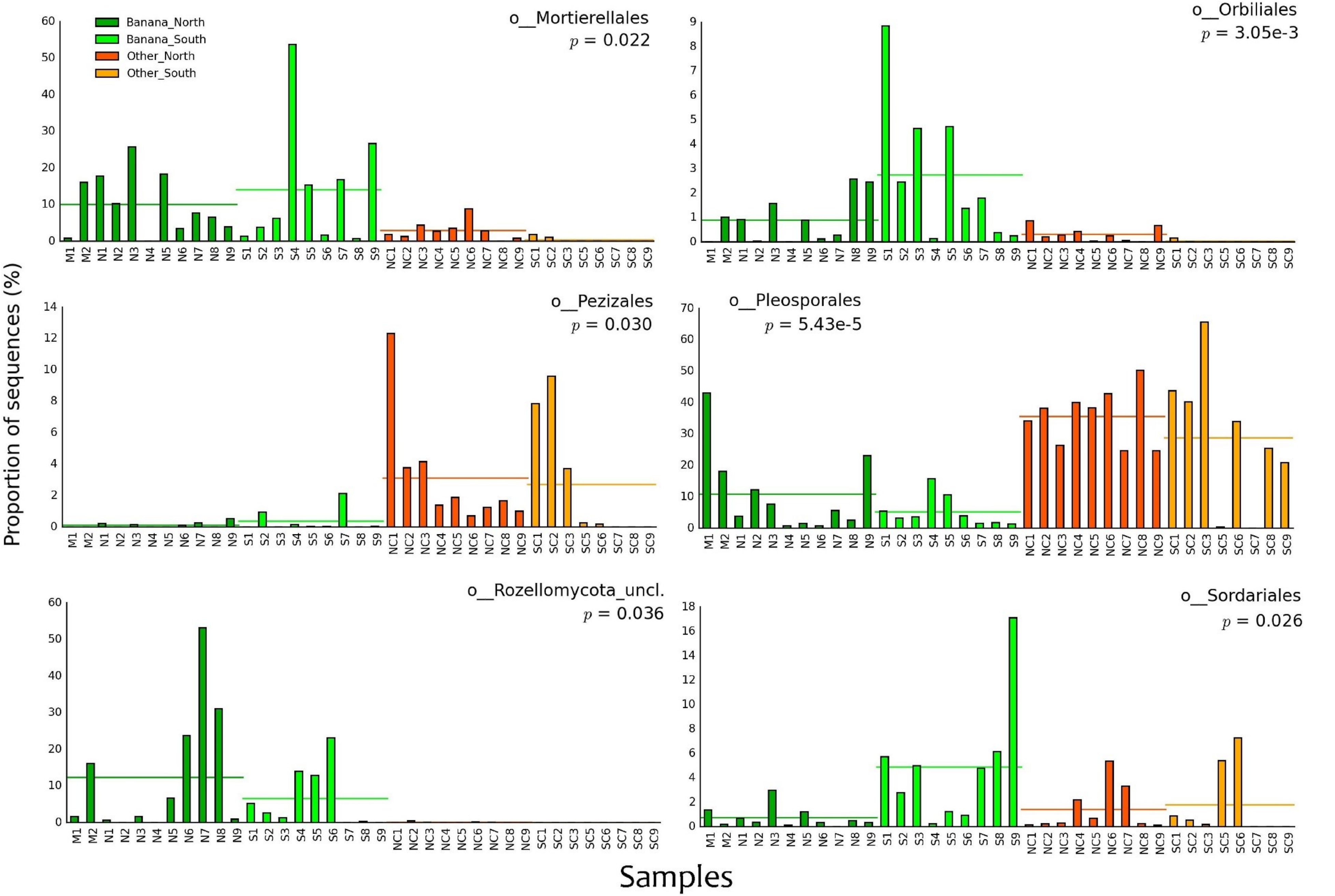

Supplement: Supplementary file 11 [file Image_3.pdf]
