# Supplementary material for: Rhizosphere 16S-ITS Metabarcoding Profiles in Banana Crops Are Affected by Nematodes, Cultivation, and Local Climatic Variations
Source: Front Microbiol. 2022 Jun 9;13:855110. doi: 10.3389/fmicb.2022.855110 (PMC9218937; doi:10.3389/fmicb.2022.855110)

Supplementary Figure 4. Most represented fungal families based on ITS sequence data, in relation to the presence/absence of plants (A), age of crops (B), density levels of predatory (C) or free living nematodes (F), banana germplasm (D) and location of farms (E). Nematode densities (specimens · 100 cc soil $^{-1}$, all stages) were classified as high (H), low (L), medium (M) and very high (VH) based on overall means (see Supplementary Table 6 for ranges). Plots produced with R library *mctoolsr*.

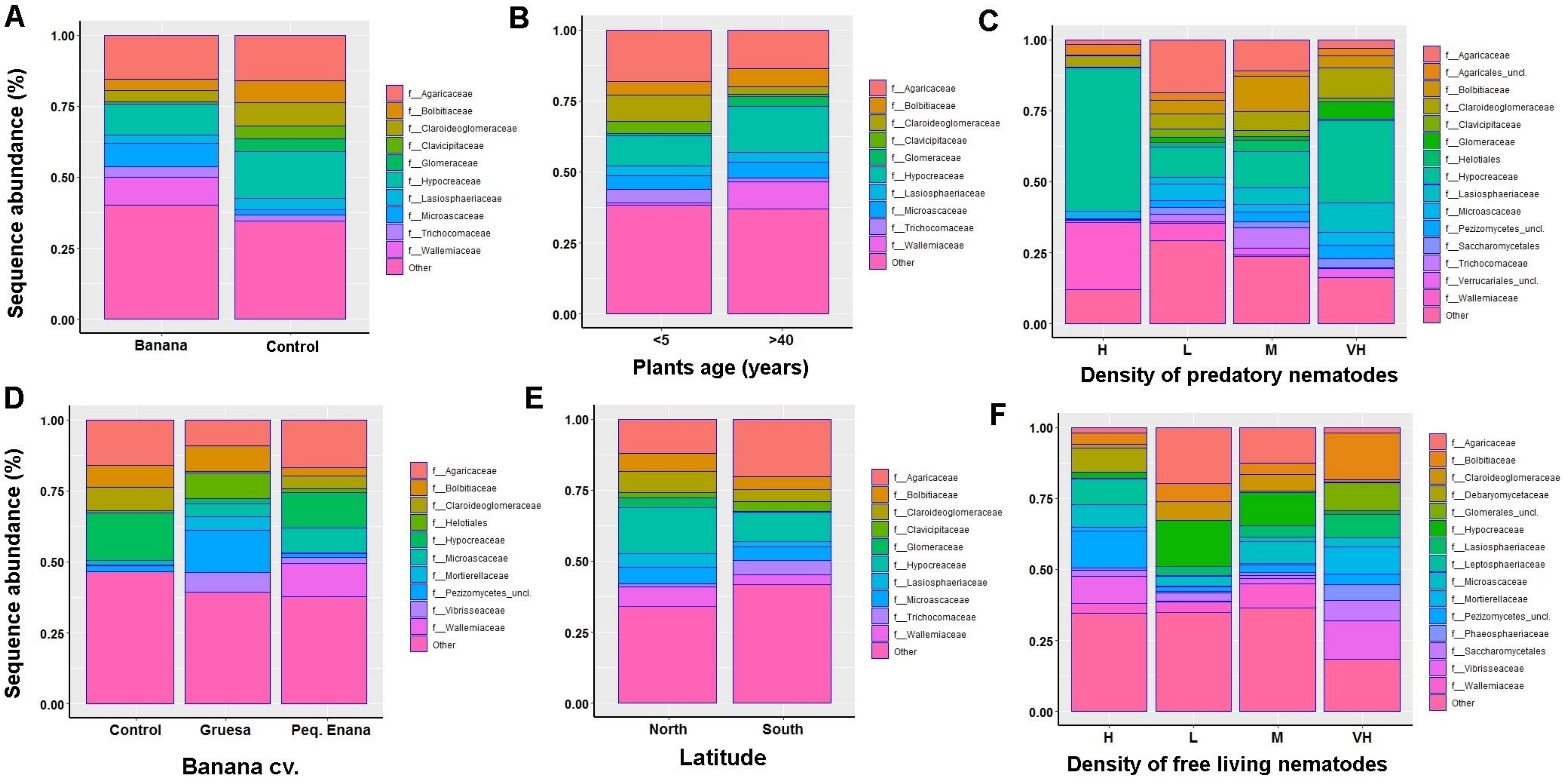

Supplement: Supplementary file 12 [file Image_4.pdf]
