# Supplementary material for: Rhizosphere 16S-ITS Metabarcoding Profiles in Banana Crops Are Affected by Nematodes, Cultivation, and Local Climatic Variations
Source: Front Microbiol. 2022 Jun 9;13:855110. doi: 10.3389/fmicb.2022.855110 (PMC9218937; doi:10.3389/fmicb.2022.855110)

Supplementary Figure 5. ITS sequence proportions of plant pathogenic fungi in banana and other adjacent control samples, shown by crop and latitude.

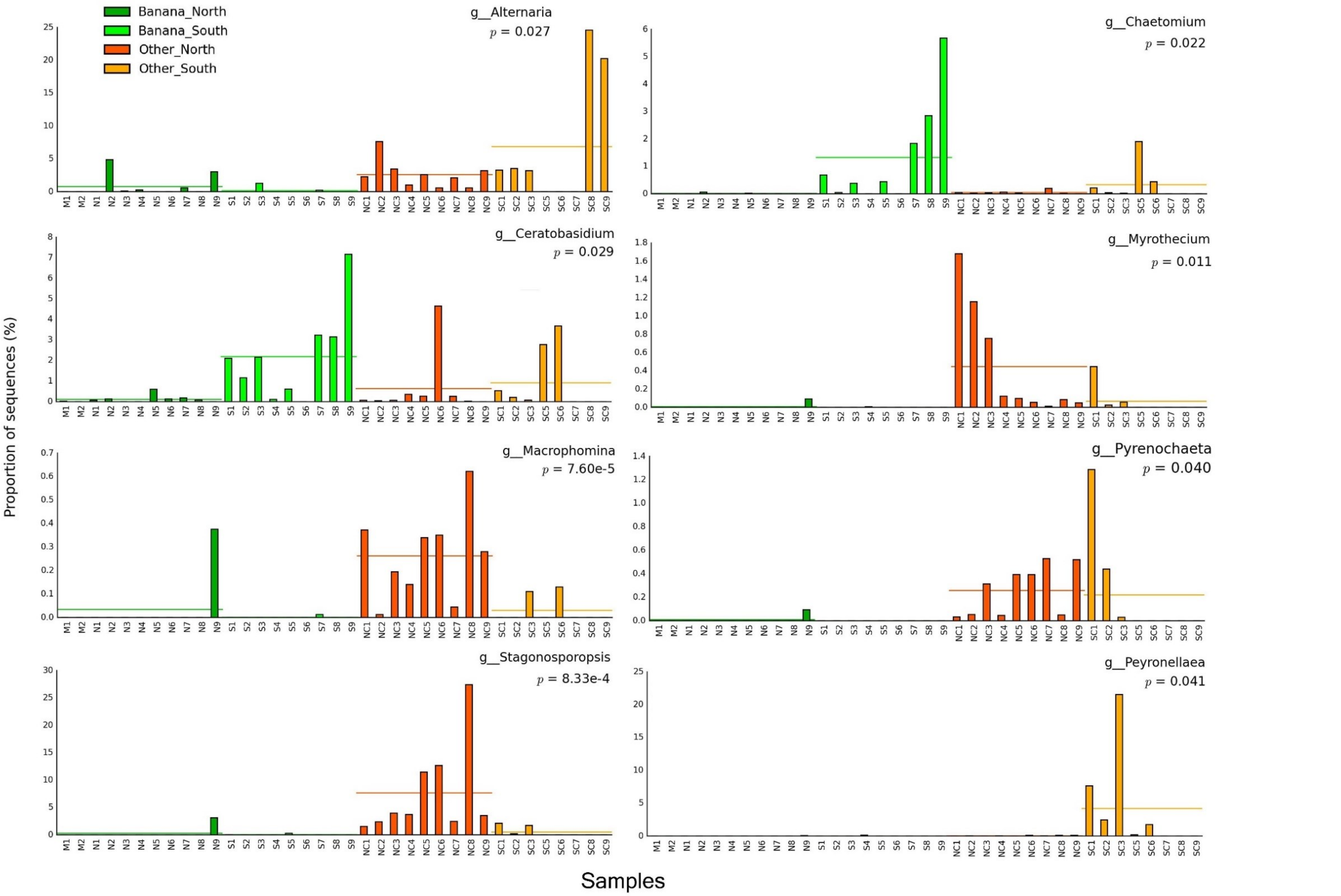

Supplement: Supplementary file 13 [file Image_5.pdf]
